# Toward Sustainability: Intensification of Light‐Driven Whole Cell Biocatalysis

**DOI:** 10.1002/cbic.70320

**Published:** 2026-04-22

**Authors:** Lenny Malihan‐Yap, Pablo Domínguez de Maria, Robert Kourist

**Affiliations:** ^1^ Institute of Molecular Biotechnology Graz University of Technology Graz Austria; ^2^ Sustainable Momentum, SL Las Palmas de Gran Canaria Spain

**Keywords:** cyanobacteria, immobilization, photobiocatalysis, reactor design, sustainability

## Abstract

Photobiocatalysis with photoautotrophic whole cells has demonstrated strong potential for producing chiral molecules and platform chemicals using sustainable inputs such as light, water and CO_2_ under mild reaction conditions. Coupling enzymatic transformations directly to natural photosynthesis enables higher atom efficiency compared with heterotrophic systems. However, large‐scale application remains challenging, particularly due to light attenuation in photobioreactors. In this review, we summarize recent advances in whole‐cell photobiotransformations with emphasis on process conditions. We also discuss strategies for intensifying photobiocatalysis through improved reactor design and new immobilization materials, along with developments in fast‐growing photoautotrophic strains. Sustainability analyses indicate that organic electron donors represent only one factor influencing environmental performance, and simply replacing them with photosynthetic water splitting does not inherently yield a carbon‐negative process. Nonetheless, our calculations show that when high substrate loadings are combined with wastewater use and optimized downstream processing, photosynthesis‐driven biotechnology can offer substantial reductions in CO_2_ emissions.

## Graphical abstract

Whole‐cell, light‐driven biotransformations in photoautotrophic microorganisms offer the potential for higher atom economy by directly harnessing reducing equivalents and oxygen generated through natural photosynthesis. Current advances include the development of strains with improved growth rates and reactor designs that mitigate self‐shading, particularly at larger scales. However, these benefits must be weighed against the volume of wastewater generated during processing to ensure that the overall approach remains sustainable for industrial application.

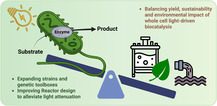

## 1 Introduction

Photobiocatalysis has emerged as a versatile route for the synthesis of organic compounds and fuels in the past decade, merging the advantages of two distinct catalytic subdisciplines, that is, photocatalysis and biocatalysis [1, 2, 3]. Using light as a benign energy source, various redox organic reactions to activate small molecules such as C–H functionalization [4, 5], cycloadditions [6], C–C/X bond formations [4, 7, 8], dehalogenations [9] and many more have been established under milder conditions outperforming their light‐independent counterparts [2]. The reported high catalytic efficiency and reaction selectivity (regio‐,chemo‐ and stereo‐) of enzyme‐based transformations under mild conditions make them attractive catalysts [10, 11]. With the advent of protein engineering, structural biology, high throughput screening and enzyme evolution technologies, biocatalysts are now seen as viable alternatives to traditional chemical catalysts used particularly in the synthesis of pharmaceutical ingredients [10, 12]. Both concepts have been extensively described and summarized in various reviews [10, 11, 13, 14]. Photobiocatalysis performed in one‐pot also significantly reduces solvent use and waste generation while improving the overall reaction efficiency, as intermediates do not need to be isolated [1].

**FIGURE 1:**
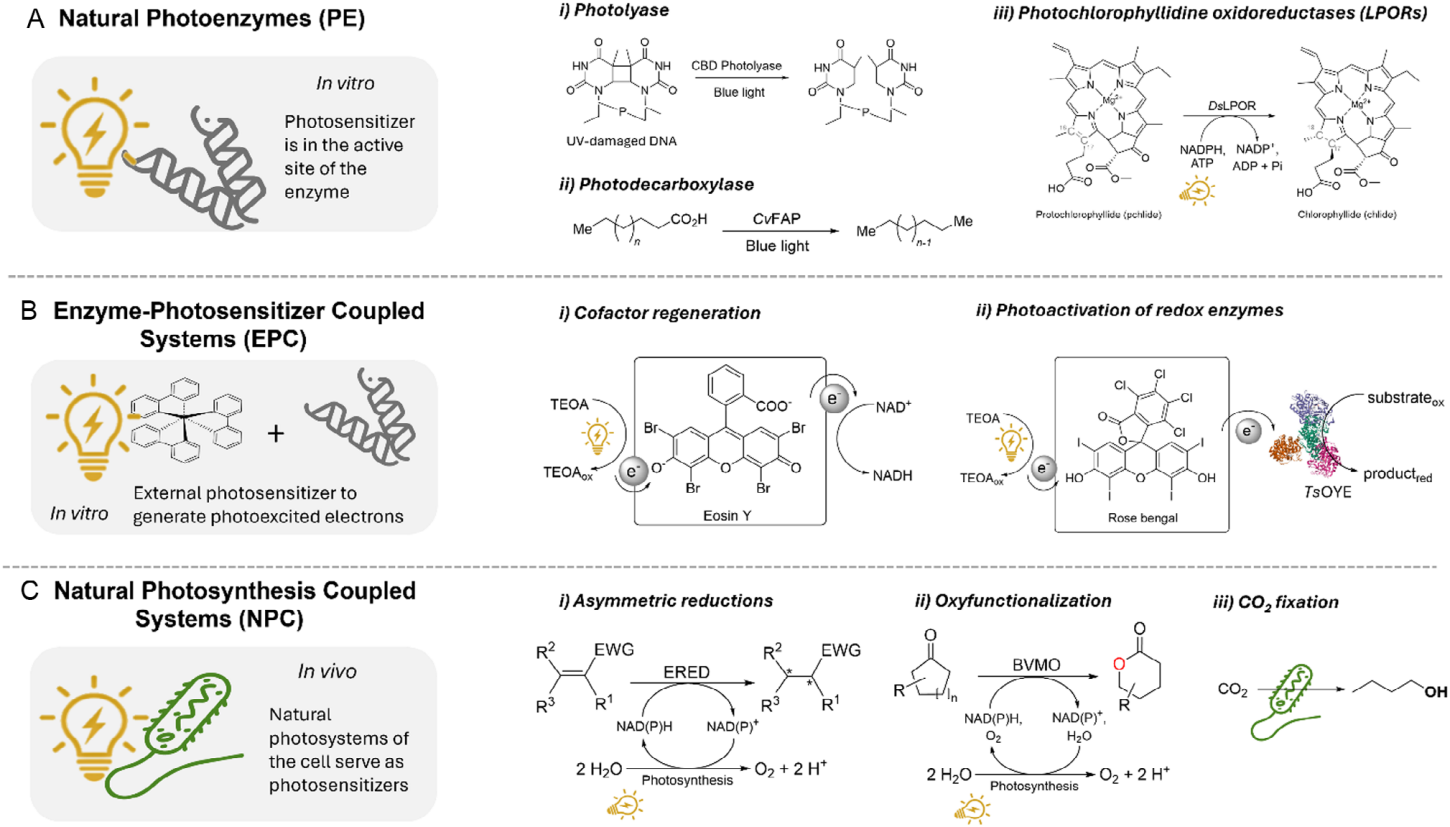
Photobiocatalytic concepts including several examples. Natural photoenzymes such as photolyases [15], photodecarboxylases [16, 17, 18] and photochlorophyllidine oxidoreductases [19] have the photosensitizer in their active sites. In EPC, an external photosensitizer is required to generate photoexcited electrons to regenerate the cofactor [20] indirectly driving the enzymatic reaction. In other instances, they directly activate the redox enzymes through their flavins. In NPC, the photosystems of the cells serve as photosensitizers prompting reactions to be performed in vivo [3].

To date, photobiocatalytic concepts can be classified into three subdisciplines: (a) Photoenzymes (PE); (b) enzyme‐photosensitizer coupled systems (EPC); and (c) natural photosynthesis coupled systems (NPC) (Figure 1) [1, 11, 21, 22]. Natural PEs such as photolyases [15], photodecarboxylases [16, 17, 18] and photochlorophyllidine oxidoreductases (LPORs) [19] have their photosensitizers in the active site of the enzyme. In contrast, EPC systems require an external photosensitizer to drive the redox transformation, such as porphyrins including tetra(4‐carboxyphenyl) porphyrin [20], or rely on direct photoexcitation of the enzyme's flavin cofactor [23]. In addition, organic dyes such as Rose Bengal have been shown to transfer excited electrons to redox enzymes [24]. Other classes of photosensitizers that have successfully driven enzymatic reactions include semiconductor‐based quantum dots [25] and organometallic complexes [26]. A review from Özgen et al. (2021) [22] summarizes various light‐driven cascades combining photo‐chemocatalytic and biocatalytic transformations.

Within this broader landscape, natural photosynthesis in microalgae or cyanobacteria offers a promising avenue for systems engineering, as the absorbed light energy could be primarily directed toward biochemical reactions rather than structural biomass formation, in contrast to higher plants. Consequently, the enzymes involved in photosynthesis, that is, photosystem II (PSII) and photosystem I (PSI) are one of the few known photocatalytic enzymes together with the aforementioned PEs: photolyases [15], protochlorophyllide reductase [19], and photodecarboxylase [16]. Among these, the photosensitizer in NPC is a part of the cell's photosystem. Hence, reactions should be performed in vivo [3].

**FIGURE 2:**
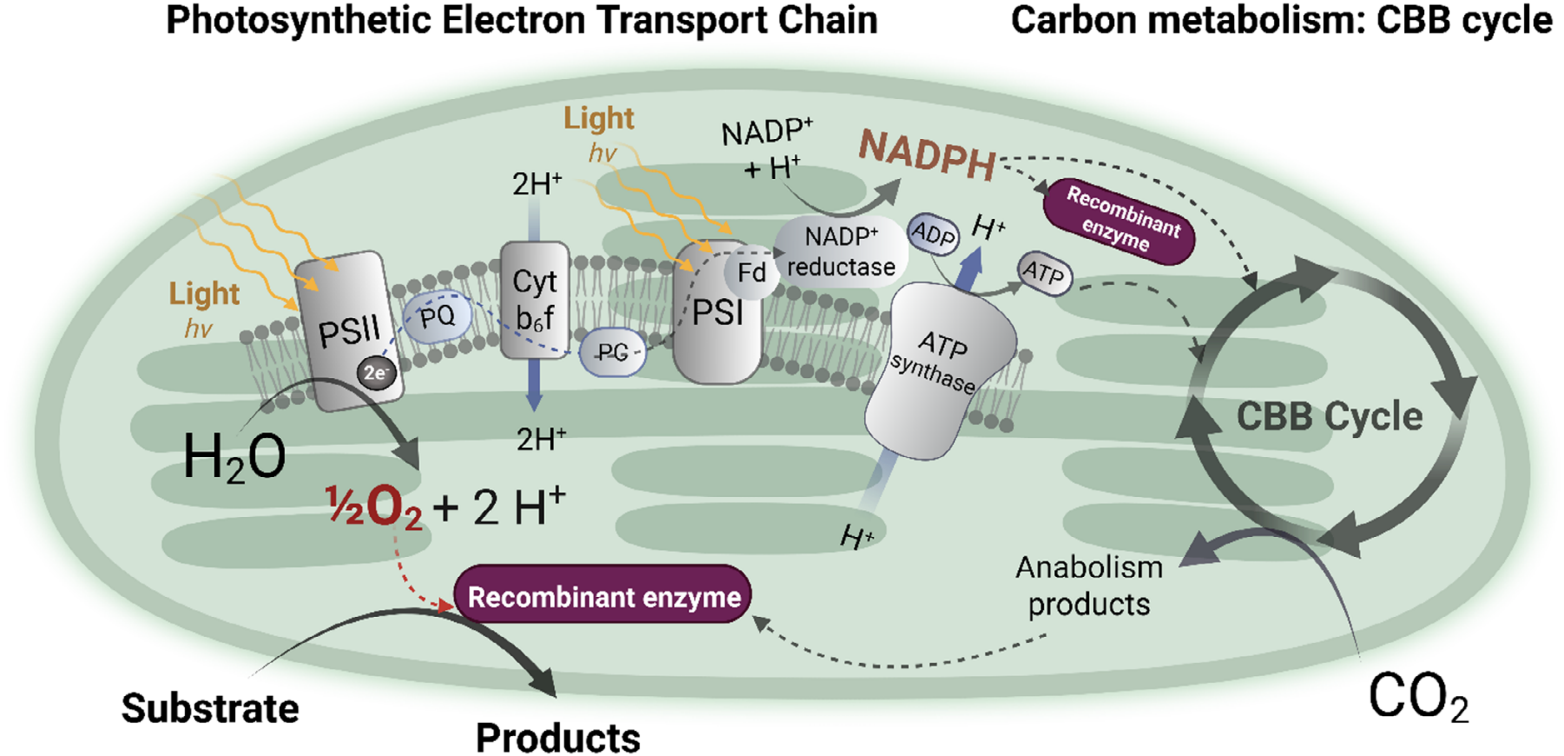
Simplified diagram of the linear electron transport in the cyanobacterium *Synechocystis* sp. PCC 6803 and the Calvin‐Benson‐Bassham cycle. Light energy is harvested at PSII, where water is oxidized to release oxygen and electrons. Photosynthetic electrons are then shuttled to PSI via cytochrome *b_6_f,* finally reducing ferredoxin using FNR and regenerating NADPH. This creates a proton gradient across the thylakoid lumen, driving the production of ATP from ADP by ATP synthase.

Light‐driven biotransformations in NPC have been successfully demonstrated in cyanobacteria and microalgae, particularly *Synechocystis* sp. PCC 6803 (hereafter *Synechocystis* sp.) and *Chlamydomonas reinhardtii* (hereafter *C. reinhardtii*), as summarized in various reviews [21, 27, 28, 29, 30]. During oxygenic photosynthesis, reducing equivalents in the form of reduced ferredoxin (Fd_red_) and reduced nicotinamide adenine dinucleotide phosphate (NADPH) are regenerated (Figure 2). In PSII, water is oxidized to release protons, oxygen, and electrons, which are transferred through the photosynthetic electron transport chain (PETC) to PSI, ultimately reducing ferredoxin [31, 32]. The electrons are then supplied from Fd_red_ to Fd‐NAD(P)H oxidoreductase (FNR) for NADPH formation, which, together with adenosine triphosphate (ATP) generated via the proton motive force, fuels CO_2_ fixation in the Calvin‐Benson‐Bassham cycle [33].

Photosynthetic reducing power has been harnessed to drive heterologously expressed oxidoreductases in *Synechocystis* sp., first demonstrated by Köninger et al. [34]. Alkene reductions mediated by the ene‐reductase YqjM achieved specific activities up to 150 U g_DCW_^−1^ through PETC engineering [35]. Similarly, expression of a Baeyer–Villiger Monooxygenase (BVMO) gene in *Synechocystis* sp. enabled a tenfold increase in activity compared to the widely known cyclohexanone monooxygenase (CHMO) from *Acinetobacter* sp [36]. Subsequent studies reported specific activities up to 60 U g_DCW_^−1^ for cyclohexanone oxidation using a BVMO from *Acidovorax* sp. [37]

Apart from these examples, several genes of oxidoreductases were expressed in cyanobacteria as well as microalgae, which demonstrated that coupling reaction systems to natural photosynthesis is a viable route for the synthesis and production of chemicals [21, 27, 28, 38]. Furthermore, photoautotrophic microorganisms have been exploited in bio‐hydrogen production using oxygen‐tolerant hydrogenases [39, 40]. In a recent study, chimeric NAD^+^‐reducing [NiFe]‐hydrogenases were developed to combine both O_2_ tolerance with ferredoxin compatibility toward photosynthesis‐coupled H_2_ production [41].

Despite these advances in synthetic biology and PETC engineering in photoautotrophic microorganisms, most of the reactions have been performed in small‐scale so far. Light‐driven biotransformation in large‐scale cylindrical illuminated vessels in volumes up to five liters [37, 42, 43, 44] suffered from significantly decreased specific activity and volumetric productivity [37], which is mainly attributed to light attenuation in the reactors, leading to a cell‐density limitation for biocatalytic reactions in light‐absorbing cells, requiring further intensification efforts to circumvent that challenge.

In this review, we want to highlight various reactor systems used in cyanobacterial or microalgal biotransformations to illustrate how the different geometries can affect product formation rates, volumetric productivity as well as space‐time‐yield (STY) and the overall process design. As will be discussed in the succeeding sections, light availability plays a major role in the upscaling of cyanobacterial/microalgal biotransformations. Hence, reactor systems, either in suspension or immobilized, aiding to alleviate the “self‐shading” phenomena in light‐driven reactions will be described. The concept and advantages of whole cell photobiotransformation are illustrated and a short recapitulation of small‐scale batch reaction systems are presented to be able to compare to large‐scale batch reactors and, finally to continuous and/or immobilized systems. Furthermore, materials, particularly polymers, utilized to encapsulate photoautotrophic microorganisms will be described and expanded. In a traversal way, the associated (un)sustainability of the current photobiocatalytic processes will be discussed to put forth thresholds and conditions from which the emerging systems could display a diminished environmental burden.

## 2 Whole Cell Photobiotransformation

Biotechnological production either employs fermentation or biotransformation, wherein the former uses cellular metabolism to convert a carbon source into a product of choice while the latter involves conversion of a substrate into a final product via single or multiple enzymatic steps [27]. To tap into the pool of reducing equivalents provided during photosynthesis, reactions are mostly performed in vivo using whole cells. Given the highly complex structure and specific reactions of the photosystems, reactions in vitro would prove unlikely [2].

Whole‐cell biocatalysts provide multiple benefits compared to crude or purified enzymes, including the inherent supply of necessary cofactors for the reaction, being cost‐effective [45, 46]. Moreover, the cell wall provides protection against harsh reaction conditions or, in some cases, when unconventional solvent systems (e.g., deep eutectic solvents, ionic liquids, and surfactants) are used [45, 47, 48].

**FIGURE 3:**
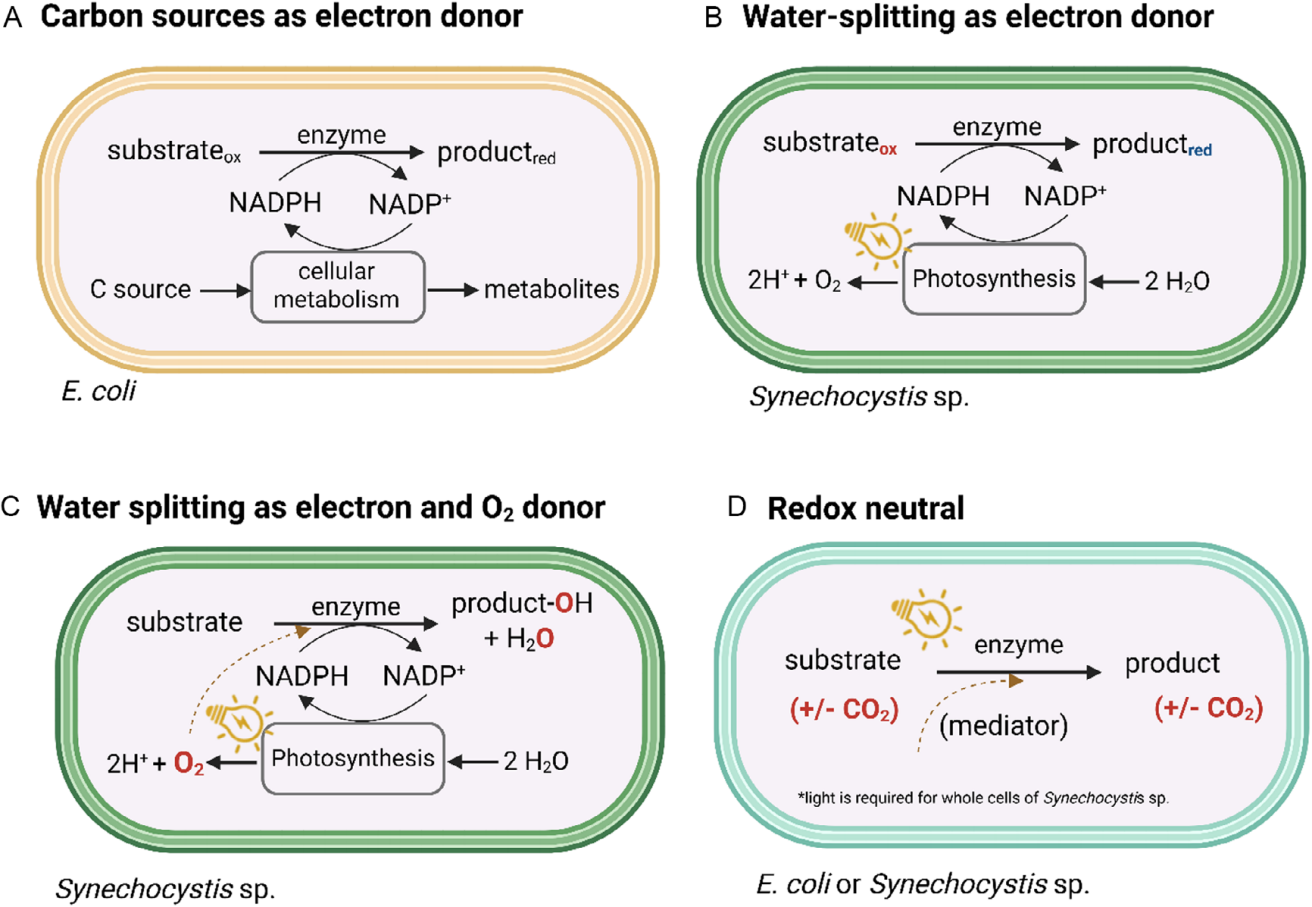
Whole cell biotransformation mediated by oxidoreductases in heterotrophic *E. coli* and photoautotrophic *Synechocysti*s sp. To regenerate the NADPH cofactor in *E. coli*, external carbon sources such as glucose or glycerol are added to the medium. In comparison, using autotrophic *Synechocystis* sp., only water is required to supply electrons during photosynthesis, regenerating NADPH and releasing oxygen. In some redox biotransformations, such as oxyfunctionalization and hydroxylations, photosynthetic oxygen is also utilized in the reaction.

To take advantage of the photosynthetic reducing power, genes encoding cofactor‐dependent enzymes are frequently introduced into cyanobacteria or microalgae. Unicellular strains such as *Synechocystis* sp. and *C. reinhardtii* are commonly employed for whole‐cell biotransformations, either using their native metabolic pathways or through genetic modification [21, 27, 49, 50]. However, it should be taken into consideration that genetic manipulation of *C. reinhardtii* is challenging due to its strict codon usage and low transgene expression coupled with lack of adequate molecular tools [51]. Oxidoreductases, in particular, are studied in cyanobacterial hosts due to their reliance on NAD(P)H. Figure 3 illustrates whole‐cell biotransformation in two microbial hosts and highlights the corresponding host types and their electron sources. Using whole cells of heterotrophic *Escherichia coli* (hereafter *E. coli*), rich carbon sources such as glucose or glycerol are supplemented in the medium to regenerate NAD(P)H. Glucose undergoes phosphorylation to glucose‐6‐phosphate during glycolysis and enters the pentose phosphate pathway to regenerate NADPH. In contrast, glycerol is converted to dihydroxyacetone phosphate via the dihydroxyacetone phosphate (DHAP) pathway, which then yields NADPH via the citric acid cycle [52, 53]. However, it is still unknown how much electrons from these carbon sources are directed toward whole‐cell biotransformation. Moreover, considerable portions of the electrons are directed toward respiration (i.e., biomass formation). Hence, an excess amount of glucose is added to compensate for metabolic losses, which greatly reduces the atom economy (AE) of the process [46].

In photoautotrophic microorganisms such as cyanobacteria and microalgae, reducing equivalents in the form of NAD(P)H are regenerated in the PETC, which are then subsequently utilized in the enzymatic reaction particularly oxidoreductases. In parallel, water splitting in the PETC produces O_2_ (Figure 3C), which is released directly in to the medium and can be used in oxygen‐dependent biotransformations, as shown previously [54, 55]. Lastly, a redox‐neutral reaction is described, such as those performed by decarboxylases, which can either be cofactor‐free or dependent on some coenzymes [56].

Photocatalytic carboxylation, which uses CO_2_ as a reagent without additional oxidants or reductants, reduce the overall energy input needed for CO_2_ conversion [57]. By transforming waste CO_2_ into valuable products under mild conditions, these reactions lower the energy cost of carbon capture and utilization. This makes the process more cost‐effective and contributes to net CO_2_ savings by turning it into valuable products.

Table 1 shows several redox biotransformations performed in whole cell microbial hosts, *E. coli* and *Synechocystis* sp. Here, AE, which is an important parameter in screening reactions, is emphasized. An efficient reaction ideally exhibits 100% AE, indicating that all atoms from the reactants are quantitatively incorporated into the desired product, without byproduct generation [70, 71]. Another parameter termed reaction mass efficiency (RME) is coined to provide a fuller picture of the reactants integrating the yield and stoichiometry to the equation. In contrast to AE, RME also considers the yield and stoichiometry of each of the reactants, which is calculated as the mass of the product over the total masses of the reactants [70]. Apart from RME, other metrics such as carbon efficiency, mass intensity and mass productivity can be calculated to predict the sustainability of the reaction.

**TABLE 1:** Comparisons of whole‐cell redox biotransformations using heterotrophic *E. coli* and autotrophic *Synechocystis* sp.

Entry	Strain	Gene expressed	Electron donor	Substrate	Product (s)	Sp. Act., U g_DCW_^−1^ a	Vol. Prod., g L^−1^ h^−1^	AE^b^, %	RME^c^, %	Ref.
**(a) Ene‐reduction**										
1	*Synechocystis* sp.	Ene reductase YqjM from *B. subtilis*	Water	2‐methylmaleimide, 50 mM	2‐methylsuccinimide, 50 mM	56.1	1.0	88	76	[46]
2	*E. coli* BL21 (DE3)	Ene reductase from *Nostoc* sp. PCC 7120	Formate, 450 mM	(*R*)‐carvone, 300 mM	(*2R*, *5R*)‐dihydrocarvone, 287mM	28.4	8.7	78	70	[58]
3	*E. coli* BL21 (DE3)	Ene reductase from OYE3	Glucose, 4 mM	(*R*)‐(‐)‐massoia lactone, 3 mM	(*R*)‐(+)‐δ‐decalactone, 3 mM	7.5^e^	3.1^e^	49	34	[59]
**(b) Hydroxylation**										
4	*Synechocystis* sp.	Cytochrome monooxygenase CYP110D1 from *Nostoc* sp.	Water	Testosterone, 1 mM	15β‐hydroxytestosterone, 0.67 mM	1.0	0.03	>99	71	[60]
5	*E. coli* C43 (DE3)	Cytochrome monooxygenase CYP110D1 + Pdx + PdR^d^	Glucose, 30 mM	Testosterone, 1 mM	15β‐hydroxytestosterone, 0.42 mM	0.5	0.13	65	2	[60]
6	*Pseudomonas putida* S12	Cytochrome monooxygenase CYP106A2 + Fdx + FdR	Glucose, 27 mM	Testosterone, 0.1 mM	15β‐hydroxytestosterone, 0.01 mM	n.d.	4.7 × 10^−5^	65	0.04	[61]
7	*E. coli* BL21	Cytochrome monooxygenase CYP106A2 + Adx + AdR + ADH	Isopropanol, 400 mM	Testosterone, 0.25 mM	15β‐hydroxytestosterone, 0.10 mM	3.2 × 10^–3^ e	1.2 × 10^−3^	88	0.13	[62]
**(c) Oxyfunctionalization**										
8	*Synechocystis* sp.	Alkane monooxygenase from *P. putida*	Water	Nonanoic acid methyl ester, 10 mM	ω‐Hydroxynonanoic acid methyl ester, 65 µM	1.5	3.7 × 10^−2^	99	0.71	[54]
9	*Synechocystis* sp.	Cyclohexanone monooxygenase from *Acinetobacter* sp.	Water	Cyclohexanone, 5 mM	ε‐Caprolactone, 2.5 mM^e^	2.3	n.d.	98	n.d.	[63]
10	*Synechocystis* sp.	Baeyer‐Villiger monooxygenase from *Burkholderia xenovorans*	Water	Cyclohexanone, 10 mM	ε‐Caprolactone, *ca.* 8.5 mM	25	0.4	98	99	[36]
11	*Synechocystis* sp.	Baeyer‐Villiger monooxygenase from *Acidovorax* sp. CHX100	Water	Cyclohexanone, 5 mM (0.25 M)^f^	ε‐Caprolactone, n.r.	60^f^ 30^g^	0.2^e^	98 77^h^	n.d.	[37]
12	*E. coli* TOP10	Cyclohexanone monooxygenase from *Acinetobacter* sp.	Glycerol, 109 mM	Bicyclo [3.2.0]hept‐2‐en‐6‐one, 0.3 g g_adsorbent_^−1^	Regio‐isomeric lactones^i^, 9 g L^−1^	87^e^	0.6	53	n.d.	[64]
13	*E. coli* BL21 (DE3)	Cylohexanone monooxygenase from *Acinetobacter* sp.	Glucose, 30 mM	Cyclohexanone, 30 mM (fed 3x, total 90 mM)	ε‐Caprolactone, 69 mM	18	0.8	37	60	[65]
**(d) (De)carboxylation**										
14	*Synechococcus* sp. PCC 11 901	Phenolic decarboxylase from *B. subtilis*	—	Ferulic acid, 80 mM	4‐Vinyl guaiacol, *ca.* 80 mM	7^d^	0.2	77	76	[66]
15	*Lactobacillus brevis* NCL912	Glutamic acid decarboxylase	—	Glutamate, 400 mM (fed 3x, total 1200 mM)	γ‐Aminobutyric acid, 1006 mM	n.d.	2.2	70	53	[67]
16	*Bacillus megaterium* PYR2910	Pyrrole decarboxylase	—	Pyrrole, 400 mM	Pyrrole‐2‐carboxylate, 325 mM	n.d.	3.0	66	14	[68]
17	*E. coli* BL21 (DE3)	Salicylic acid decarboxylase	—	*m*‐Aminophenol, 200 mM	*p*‐Aminosalicylic acid, 140 mM	800	2.4	73^j^	5	[69]

a 1 Unit (U) of activity corresponds to 1 µmol min^−1^ product formed.

b Atom economy = (MW_product_/Total MW_substrates_).

c Reaction Mass Efficiency = Mass of product/Total Masses of Substrates [70].

d Putidaredoxin + putidaredoxin reductase.

e Calculated from given data.

f Small‐scale.

g CSTR 2L.

h With aeration.

i Regio‐isomeric lactones derived from bicyclo [3.2.0]hept‐2‐en‐6‐one oxidation: (‐)1(*S*), 5(*R*) 2‐oxabicyclo [3.3.0]oct‐6‐en‐3‐one and (‐)1(*R*), 5(*S*) 3‐oxabicyclo [3.3.0]oxabicyclo [3.3.0]oct‐6‐en‐2‐one.

j KHCO_3_ was used as CO_2_ source.

Abbreviation: n.d. = not determined.

The AE of cyanobacterial‐mediated redox biotransformations is higher compared to those mediated by *E. coli* reaching *ca.* 99% particularly in hydroxylation and oxidation reactions. This is mainly attributed to its inherent cofactor regeneration in the PETC as well as the released oxygen, which in turn are utilized in the reaction [54, 55, 60]. Whole cells of heterotrophic *E. coli*, either growing or resting, require additional carbon sources in the form of glucose or glycerol to regenerate NADPH. Asymmetric ene‐reduction catalyzed by the ene‐reductase (ER) YqjM produced in *Synechocystis* sp. (Table 1, Entry 1) showed an AE of 88%. In comparison, when ERs are produced in *E. coli*, AE can decrease up to 49% when glucose is supplemented (Table 1, Entry 3). By coexpressing the genes of a formate dehydrogenase with an ER from *Nostoc* sp. in *E. coli*, the AE is relatively high (78%, Table 1, Entry 2) due to the small molecular weight of formate. A high RME was also reported for the same reaction due to a higher product formation rate and consequently specific activity by implementing an NADH‐accepting ER mutant. Termed as Loop 1,2a‐FDH, the mutant was constructed by swapping loop regions of the *Nostoc*ER1 for the corresponding regions of two NADH‐favoring ERs [58, 72]. Compared to wild type *Nostoc*ER1‐FDH, the mutant displayed a 2.1‐fold increase in initial product formation rate (2.8 mmol h^−1^ g_DCW_^−1^) corresponding to an STY of 57.4 mmol L^−1^ h^−1^ (or 8.7 g L^−1^ h^−1^). The NADH concentration in glucose‐grown *E. coli* cells are 20‐fold higher as compared to NADPH, which makes utilization of the former more effective [73]. Moreover, the larger variety of NAD(H)‐regenerating enzymes [74] makes this mutant more economically attractive.

Oxygen derived in PSII is also an invaluable substrate for various organic reactions such as hydroxylation and oxyfunctionalization. Released homogeneously in the liquid medium, photosynthetic oxygen overcomes the gas–liquid interface associated with the utilization of gaseous oxygen [54] and eases its delivery in continuous flow systems [55]. Cyanobacterial redox biotransformations involving oxygen often outcompetes its heterotrophic counterparts with AE almost at 100% efficiency (Table 1, Hydroxylation and Oxyfunctionalization entries). In contrast, requiring an excess of sacrificial cosubstrates in the form of either glucose or glycerol to regenerate NAD(P)H significantly decreases the AE. Notably, in *E. coli*‐mediated oxyfunctionalization, AE can decrease up to 37% (Entry 13) due to reliance in NAD(P)H and oxygen. It should be noted that at higher reactor volumes (i.e*.*, Continuous Stirred Tank Reactors), oxygen (rate = 2 L min^−1^) is also supplied even when utilizing cyanobacteria, thereby reducing the AE from 98% to 77% (Table 1, Entry 11).

However, despite having low AE, RMEs using *E. coli*‐mediated oxyfunctionalization can be increased by employing a high initial substrate concentration via fed‐batch feeding [65] (Table 1, Entry 13). This can be coupled with an efficient downstream processing (DSP) [75], for example, in situ substrate feeding/product removal (SFPR). A “two‐in‐one” resin‐based SFPR pioneered by Vicenzi et al. [76] was applied containing the substrate acting as a reservoir while continuously removing the product. This allowed volumetric productivities up to 1.2 g L^−1^ h^−1^ from a substrate loading of 25 g L^−1^, which suggests a promising metric for sustainable processes as well [77]. Similar strategies could also be applied for cyanobacterial/microalgal biotransformations to increase substrate loading with subsequent product removal. In this field, such strategies could mitigate toxicity of either the substrate or the product particularly in living whole‐cell biotransformations.

Apart from oxidoreductions, cyanobacterial whole‐cell biotransformations employing lyases were recently established. In 2015, we reported the heterologous production of two cofactor‐free enzymes, a decarboxylase and an esterase in cyanobacteria from CO_2_ and a nitrogen source [78]. Rudroff et al. [66] elaborated this concept by expressing the gene of a cofactor‐free decarboxylase in the fast‐growing cyanobacterium *Synechococcus* sp. 11901, and using the enzyme in a whole‐cell biocatalyst for decarboxylation of ferulic acid. Herein, lyases neither require photosynthetic electrons nor photosynthetic oxygen, potentially allowing operations at higher cell‐densities. Rather than in the biotransformation reaction itself, the great advantage of this approach lies in the preparation of the biocatalyst, where edible resources as growth substrate are substituted by carbon dioxide and an inorganic nitrogen source. These potential savings, however, should be compared with the illumination energy and the longer (envisioned) cultivation times of many cyanobacterial strains. The use of fast‐growing cyanobacteria addresses this issue and has great potential to improve the environmental footprint of whole‐cell biotransformations. Importantly, the whole‐cell decarboxylation does not require illumination during the biotransformation, which allows the utilization of established bioreactors. However, efficient supply of electrons and oxygen by photosynthesis requires efficient illumination and poses challenges for the design of the bioreactor.

## 3 Reactor Concepts Associated to Photobiocatalysis

Photobioreactor design is a crucial aspect in achieving high volumetric productivities using whole cell biotransformation in photoautotrophic microorganisms. To move toward scalable systems, uniform and controlled light illumination should be delivered to all reactor parts [3]. Light intensity directly influences microalgal growth, achieving maximum photosynthetic activity until reaching a saturation point. However, when it falls below the optimal value, growth as well as activity are compromised [79]. In contrast, if the available light exceeds the optimum, photoinhibition may occur producing reactive oxygen species, potentially damaging the photosystems particularly PSII [32]. Hence, a balanced supply of light, together with uniform distribution along the photobioreactor, is essential to achieve optimal yields and productivities to be competitive with heterotrophic‐based biotransformations.

**FIGURE 4:**
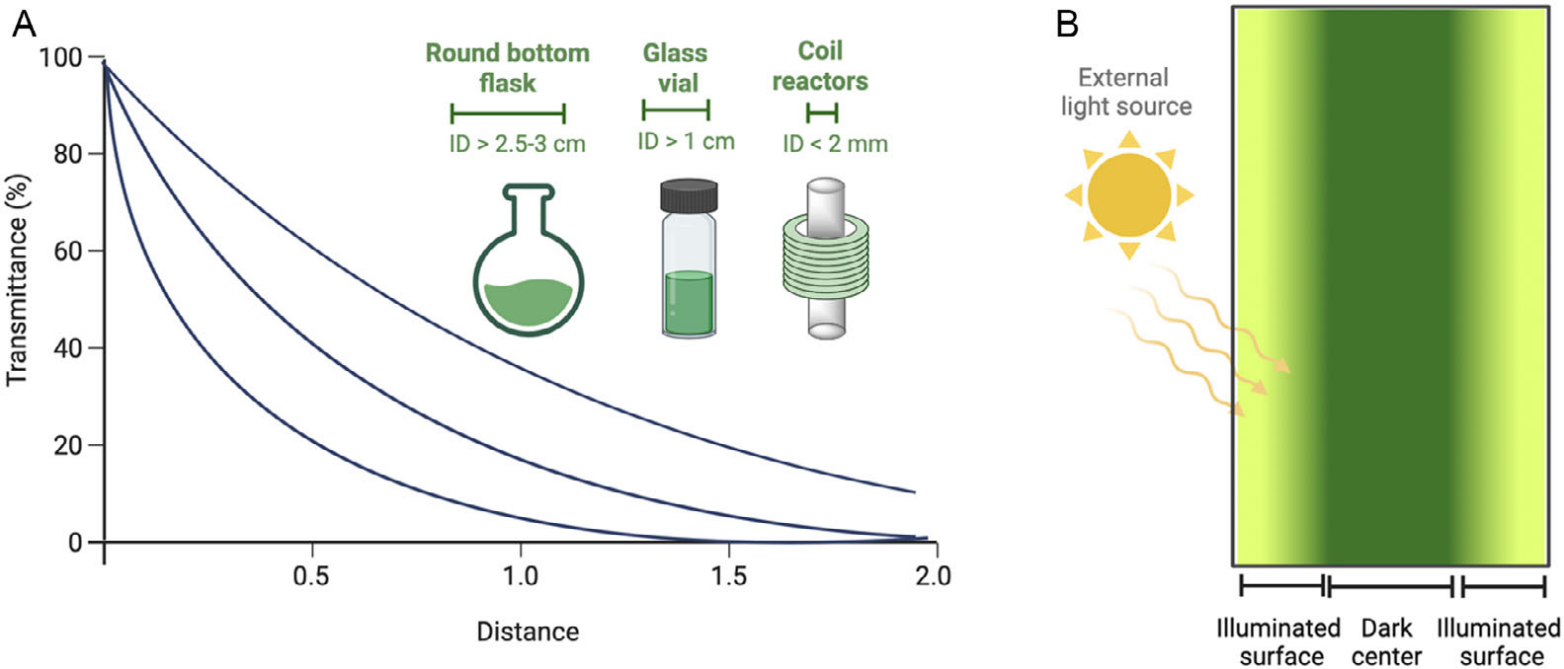
(A) Light attenuation with distance from the light source (adapted from [80]) and (B) Light distribution in a batch photobioreactor using an external light source.

Light transmittance decreases exponentially from the distance to the light source (Figure 4A) according to the Beer‐Bouguer‐Lambert law. Slow reactions and inhomogeneous irradiation are oftentimes encountered in large reaction vessels (internal diameter, ID > 1 cm) where regions in the center remain in dark, and only the vessel walls are illuminated (Figure 4B) [80]. This could be circumvented by utilizing microreactors with internal diameters in the millimeter range. Notably, continuous flow‐microreactors are gaining attention, providing several advantages over batch photobioreactors. Due to their small channels, uniform irradiation is provided, which in turn, accelerates the reaction with minimal catalyst loading. Furthermore, byproduct formation is minimized and the productivity is increased [81].

In the following sections, photobioreactors for whole cell biotransformation in photoautotrophic microorganisms will be described both for wild type and recombinant strains. Key parameters will be light intensity, reactor volume, volumetric productivities (in case of batch reactions) or STY (in continuous reactors) together with specific activity data, which are normalized either in dry cell weight mass (g_DCW_^−1^) or chlorophyll *a* mass (mg_chla_^−1^).

### 3.1 Batch Reactors

#### 3.1.1 Wild Type Strains

**FIGURE 5:**
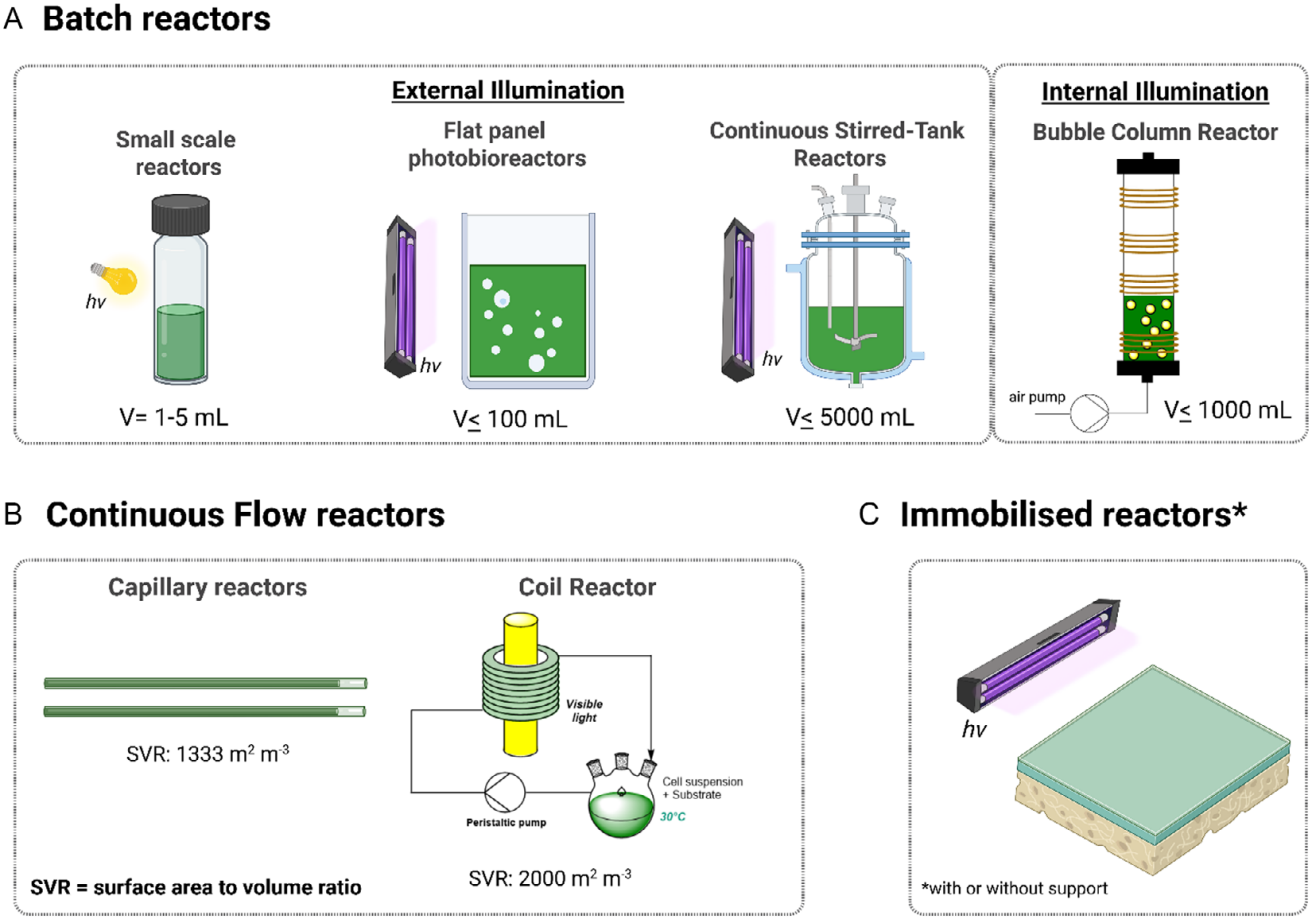
Various reactors utilized for light‐driven biotransformations in photoautotrophic microorganisms. Batch reactors with volumes ranging from 1‐ to 5000 mL are mostly externally illuminated. A bubble column reactor enabled internal illumination in dense cyanobacterial reaction, delivering 61 µmol photons m^−2^ s^−1^ at 2.4 g L^−1^ cell density [82]. Continuous reactors with high surface‐to‐volume ratio (SVR) such as film and coil are utilized to alleviate the light attenuation, especially in dense cultures. Immobilization in thin films have also been employed to increase the amount of cells in a surface area, prolong the biocatalytic activity, minimize water usage and simplify DSP [83].

As previously mentioned, a uniform light provision greatly influences the overall performance of whole‐cell biotransformations mediated by photoautotrophic microorganisms. Figure 5 shows various reactor configurations ulized for whole‐cell photobiotransformations ranging from batch to continuous reactor systems. Initial photobiotransformations were conducted using batch reactors with external illumination (Figure 5A), with reactors ranging from glass vials to photobioreactors, occupying volumes from one milliliter to 2000 liters. Table 2 lists whole‐cell biotransformations mediated by wild type strains. In contrast to recombinant strains, where the reaction is catalyzed by a specific enzyme, wild type strains often rely on one or a combination of enzymes, many of which may be unknown, representing one of the drawbacks of the latter [27].

**TABLE 2:** Light‐driven whole‐cell biotransformations performed in batch reactors using wild type cyanobacterial and microalgal strains.

Entry	Strain	Reaction	Substrate	V, mL	LI, µmol photons m^−2^ s^−1^	Cell density, g_DCW_ L^−1^	Type of PhBR	Conv., %	Vol. Prod., g L^−1^ h^−1^	Ref.
1	*Synechococcus* PCC 6911 *Synechococcus* PCC 6716 *Synechocystis* sp. PCC 6803 *Anabaena variabilis* *Anabaena oscillarioides*	Aliphatic aldeyhe‐, methyl‐ and ethyl ketone reduction	Various aldehydes and ketones	100	0.3	n.r.	n.r.	n.r.	n.r.	[84]
2	*Synechococcus elongatus* PCC 7942	Ketone reduction	2′, 3′, 4′, 5′, 6′‐Pentafluoroacetophenone	2	13.4	0.6	Culture flask	100	0.53^*^	[85]
3	*Spirulina platensis* *Anabaena* sp. *Anabaena laxa* *Aphanizomenon klebahnii* *Nodularia moravica* *Chroococcus minutus* *Merismopedia glauca* *Synechocystis aquatilis*	Ketone reduction	2′‐Hydroxychalcone 2′’‐Hydroxychalcone 4′’‐Hydroxychalcone	30 250 (prep. scale)	*ca.* 17	n.r.	Erlenmeyer flask	23 99 99	1.4 × 10^−2^ 5.88 × 10^−2^ 5.91 × 10^−2^	[86]
4	*C. reinhardtii*	Ene‐reduction	*N*‐Methylmaleimide (*R*)‐Carvone (*S*)‐Carvone 2‐Cyclopenten‐1‐one 2‐Cyclohexen‐1‐one trans‐Cinnamaldehyde	10	100	100 µg_Chl*a*_ mL^−1^	Conical flask	40^b^ 70^b^ 60^b^ 60^b^ 50^b^ 0	0.06	[49]
5	*Synechococcus elongatus* PCC 7942	Ketone reduction	(+/−) Camphorquinones	50	27	1	n.r.	92	7.67 × 10^−3^	[87]
6	*Synechococcus* PCC 7942 *Anabaena variabilis* *Nostoc muscorum*	Ketone reduction	Ethyl 4‐Chloroacetate 2′, 3′, 4′, 5′, 6′‐Pentafluoroacetophenone 2′, 3′, 4′, 5′, 6′‐Pentafluoroacetophenone	1	26 0 (dark)	1.02 and 7.72 1.73 and 8.76 1.07 and 10.86	Glass vials (gas‐tight)	9 5 10	0.03 0.02 0.02	[88]
7	*Anabaena laxa* *Anabaena* sp. *Aphanizomenon klebahnii* *Nodularia moravica* *Chroococcus minutus* *Merismopedia glauca* *Synechocystis aquatilis* *Spirulina platensis*	Ketone reduction	Chalcone	30	42	1 mg_Chla_ L^−1^	Erlenmeyer flask	>99 88 >99 >99 70 92 >99 3	5.89 × 10^−5^ 5.24 × 10^−5^ 5.89 × 10^−5^ 5.89 × 10^−5^ 4.17 × 10^−5^ 5.46 × 10^−5^ 5.89 × 10^−5^ 1.79 × 10^−5^	[89]
8	*Synechocystis* sp. PCC 6803 *Synechocystis* sp. PCC 6714 *Fischerella muscicola* UTEX 1301 *Anabaena cylindrica* IAM M1 *Plectonema boryanum* IAM M101 *Anabaena* sp. PCC 7120 *Synechococcus* sp. PCC 7942	Aldehyde reduction	Cinammaldehyde	20	160	0.19	Greiner culture flask	*ca.* 75^*^ *ca.* 45^*^ *ca.* 30^*^ *ca.* 30^*^ *ca.* 24^*^ *ca.* 45^*^ 0	5.32 × 10^−4^ 9.21 × 10^−4^ 3.13 × 10^−4^ 6.25 × 10^−4^ 3.13 × 10^−4^ 4.5 × 10^−4^ n.a.	[90]

a Calculated from initial rate of product formation.

b Based on Supporting Figure S4 in Böhmer et al. [49].

c Reported as U mg^−1^ in standard reaction conditions (50 mM Tris‐HCl pH 7.5, 30ºC, 300 rpm, 5 mM substrate).

* Estimated based on figures.

Abbreviations: n.r.‐not reported. LI – light intensity.

Nevertheless, wild type strains have been described to catalyze stereospecific reduction of prochiral ketones [85, 87, 88, 89]. The ability of cyanobacteria to reduce carbonyl compounds was first observed by Jüttner et al. [84] using *Anabaena cylindrica*. Using unicellular and filamentous cyanobacterial strains, aliphatic aldehydes were easily converted to their corresponding alcohols as well as methyl and ethyl ketones, except for butanone and octan‐4‐one even after extended periods. Reduction of alicyclic ketones was limited with low to negligible yields when cyclopentanone or cyclohexanone was added. In contrast, 2,2,6‐trimethylcyclohexanone and 3,5,5‐trimethylcyclohexanone reportedly gave higher yields, albeit no specific data was presented. Further compounds that unexpectedly gave high yields were unsaturated branched ketones and aldehydes belonging to the monoterpenes and nor‐carotenoids.

The reductions in cyanobacteria are usually catalyzed by ubiquitous NAD(P)H‐dependent alcohol dehydrogenases (ADHs). In *E. coli*, ADHs prefer NADH/NAD^+^ while in cyanobacteria, the phosphorylated cofactor (NADPH/NADP^+^) is favored [27]. As mentioned previously, NADPH is regenerated in the PETC which is found in the thylakoid membranes of cyanobacteria and microalgae, and its synthesis is directly correlated to the presence of light and its intensity. This was demonstrated by Nakamura and Yamanaka [85] in the asymmetric ketone reduction mediated by *Synechococcus elongatus* PCC 7942, where the reaction rate was four times higher under illumination. Furthermore, reduction of 2′, 3′, 4′, 5′, 6′‐pentafluoroacetophenone using cell free extracts (CFE) showed a preference to NADPH over NADH with 100% activity with the former. In another study, the effect of light was shown by performing biotransformations in dark supplementing glucose (10 g L^−1^) to regenerate NADPH [88]. This was performed to evaluate whether a standard STR can be utilized for asymmetric reductions by photoautotrophic microorganisms. Surprisingly, *Synechococcus* PCC 7942 was able to produce the same titers of chiral *S*‐alcohols except for ethyl 4‐chloroacetate at an initial concentration of 100 mmol L^−1^ with nearly similar enantiometric excess (93–99.8%). In any case, though, product formations were higher under light conditions.

Aromatic prochiral ketones such as (hydroxy)chalcones were also successfully converted to their corresponding alcohols [89, 91]. The biotransformations were performed using moderate light intensity (17–40 µmol photons m^−2^ s^−1^) in culture flasks. Eight cyanobacterial strains were tested for the biotransformation of chalcone to dihydrochalcone [89] with four strains showing >99% conversions to the desired product. However, some of the strains yielded undesired compounds, and the long reaction time (14 days) coupled with low initial substrate concentration showed low volumetric productivities.

Apart from ketone reduction, aldehyde‐ [90] and ene‐reduction [49] were also demonstrated in wild‐type microalgae and cyanobacteria. Interestingly, these reactions were performed at higher light intensities (>100 µmol photons m^−2^ s^−1^). Light plays a crucial role in the regulation of enzymes in the carbon reduction cycle particularly in higher plants. In cyanobacteria, light also influences the redox state of the cells, promoting enzymatic activities based on their cofactor preferences. When grown under constant illumination (40 µmol photons m^−2^ s^−1^), the NADP(H) to NAD(H) ratio in *Synechococcus* sp. PCC 7942 was shown to be 6.5‐fold higher [92]. Moreover, even when cultivated under dark conditions, NADP(H) concentration remains higher (3.8‐fold) as compared to NAD(H).

Old yellow enzymes (OYEs) are NAD(P)H‐dependent oxidoreductases that catalyze the stereo‐ and enantioselective reduction of α,β‐unsaturated substrates in the presence of a reduced flavin mononucleotide (FMN) through a bi–bi ping‐pong mechanism [49, 93]. The requirement for the expensive cofactor NADPH prompted whole‐cell ene‐reductions as shown by Tischler et al. [94] in *E. coli* or by Köninger et al. [34] in recombinant *Synechocystis* sp. Several members of OYEs were characterized in the eukaryotic microalgae *C. reinhardtii*, allowing C=C bond reduction of various substrates, including (*R*)‐carvone. The product (2*R*,5*R*)‐dihydrocarvone is an important intermediate in natural products synthesis and a precursor for antimalarial drugs. Microalgal cells were able to convert various substrates, including (*R*)‐carvone with stereoselectivities comparable to those of *E. coli* expressing the ene‐reductase FOYE‐1 gene, as previously reported [94].

The importance of light availability is underscored by observations that wild‐type strains exhibit reduced activity when reactions are conducted in the dark [85, 90]. In addition, performing reactions in the absence of light has been shown to decrease selectivity [88].

In Table 2, it can also be observed that the cell densities used for light‐driven biotransformations do not exceed 1 g_DCW_ L^−1^ except for the study by Havel and Weuster‐Botz (2006) [88] where a higher cell density of *ca.* 10 g_DCW_ L^−1^ was used in dark reactions. At elevated cell densities, light attenuation becomes significant due to self‐shading, as illustrated in Figure 4B, where cells absorb incident light and hinder its penetration to neighboring cells [95]. In contrast, heterotrophic‐based biotransformations can be carried out at higher cell densities (25 g L^−1^) [88] without limitations imposed by light availability, provided there is no enzyme inhibition.

For the abovementioned reactions in wild type photoautotrophic microorganisms, light intensity, reactor type as well as process design have not been optimized so far. Hence, low productivities were observed due to longer reaction time and low initial substrate concentration. As previously mentioned, reductions are primarily catalyzed by NADPH‐dependent alcohol dehydrogenases endogenous in cyanobacteria. Optimizing reactions in wild‐type photoautotrophic microorganisms may be challenging due to the pool of enzymes involved in a single reaction. Identifying and characterizing the specific enzyme responsible for a given reaction is often tedious and time‐consuming, which impedes optimization. In this regard, recombinant strains are gaining attention as whole‐cell production hosts.

#### 3.1.2 Recombinant Strains

With the advent of advanced synthetic biology techniques in photoautotrophic microorganisms specifically for the cyanobacterium *Synechocystis* sp., genes of oxidoreductases have been widely expressed in its genome to produce a variety of compounds [21, 27, 28]. Gene expression can be controlled by precisely selecting the appropriate genetic elements such as the promoter. In *Synechocystis* sp., various native and light‐inducible promoters have been utilized, with the *cpc* promoter shown to be the strongest. This promoter regulates the *cpc* operon which encodes for the proteins in the phycobilisomes [96]. Other light‐inducible promoters, such as *psbA1* and *psbA2* which control the D1 subunit of PSII have also been utilized. However, being light‐inducible, the gene expression cannot be strictly controlled. Hence, synthetic promoters such as the zinc‐inducible (P_*zia*_) [35], isopropyl β‐D‐1‐thiogalactopyranoside (IPTG)‐inducible (P_*trc10*_) [60, 97], nickel‐inducible (P_*Ni2+*_), copper (P_*Cu2+*_)‐inducible [37] and rhamnose‐inducible (P_*Rha*_) [46] were also employed. The leakiness of the P_*Cu2+*_ promoter, along with the inefficient gene expression by P_*zia*_ and P_*Rha*_, rendered these promoters unsuitable for *Synechocystis* sp.

##### 3.1.2.1 Small‐Scale

Table 3 summarizes whole‐cell biotransformations mediated by recombinant cyanobacteria or microalgae. Initial studies were performed in small‐scale reactors, particularly glass vials with volumes ranging from 1 to 5 mL with a light intensity of 150–300 μmol photons m^−2^ s^−1^. In an earlier work, CFE of *Synechocystis* sp. expressing the genes of an esterase from the thermophilic organism *Sulfulobus tokadaii* and an arylmalonate decarboxylase (AMDase) from *Bordetella bronchiseptica* showed high enantioselectivity (>99%) and yield (91%) in the de‐symmetrization of prochiral malonates [78]. Expression of the genes did not impair the growth of the cells. Esterase activity increased 1.5‐fold when light intensity was increased from 150 to 300 µmol photons m^−2^ s^−1^, likely due to higher protein production driven by the *psbA2* promoter under increased light conditions. The production of AMDase in *Synechocystis* sp. also showed that genes from mesophilic organisms can be efficiently expressed in cyanobacterium. Here, a proof‐of‐concept for the straightforward protein production in a photoautotrophic microorganism was demonstrated.

**TABLE 3:** Light‐driven whole‐cell biotransformations performed in batch reactors using recombinant cyanobacterial and microalgal strains.

Entry	Strain	Enzyme produced	Reaction	Substrate	V, mL	LI, µmol photons m^−2^ s^−1^	Cell Density, g_DCW_ L^−1^	Type of PhBR	Conv., %	Vol. Prod., g L^−1^ h^−1^	Sp. Act., U g_DCW_^−1^	Ref.
**(a) Small scale**												
1	*Synechocystis* sp.	YqjM from *B. subtilis*	Ene‐reduction	Cyclohexenone 2‐Methylcyclohexenone Ketoisophorone Cyclopentenone *N*‐Methylmaleimide 2‐Methylmaleimide 2‐Methyl‐*N*‐Methylmaleimide	1.5	150	0.4–2.7	Glass vials	70 42 57 99 94 99 99	0.03 0.03 0.04 0.50 0.64 1.10 0.63	39 21 6 26 53 123 99	[34]
2	*Synechocystis* sp. (ΔFlv1 mutant)	YqjM from *B. subtilis*	Ene‐reduction	2‐Methylmaleimide *N*‐Methylmaleimide 2‐Methyl‐*N*‐methylmaleimide Cyclohexenone 2‐Methylcyclohexenone	1	150	0.48–2.4	Glass vials	*ca.* 90	2.04^a^ 1.74 2.64 1.55 0.26	147 107 133 101 16	[35]
3	*Synechocystis* sp.	Imine reductase IRED‐A	Imine reduction	5‐Methyl‐3,4‐Dihydro‐2H‐Pyrrole 6‐Methyl‐2,3,4,5‐ Tetrahydropyridine 7‐Methyl‐3,4,5,6‐Tetrahydro‐2H, azepine 3,4‐Dihydroisoquinoline 1‐Methyl‐3,4‐Dihydroisoquinoline 2,3,3‐Trimethylindole 1‐Methyl‐4,9‐Dihydro‐3H‐Pyrido[3,4‐b]indole 7‐Methoxy‐1‐Methyl‐4,9‐dihydro3H, pyrido[3,4‐b]indole	2	150	1.2–6.0	Glass vials	>99 83 >99 99 94 26 n.c. n.c.	7.4 × 10^−5^ 2.6 × 10^−5^ 1.2 × 10^−5^ 2.2 × 10^−5^ 6.1 × 10^−6^ 3.1 × 10^−6^ n.c. n.c.	21.8 8.9 5.5 10.2 3.1 1.6 n.c. n.c.	[98]
4	*Synechocystis* sp. (ΔhoxHY mutant)	YqjM from *B. subtilis*	Ene‐reduction	2‐Methylmaleimide	1	150	2.4	Glass vials	*ca.* 90	1.5 x 10^−4^	120	[99]
5	*Synechocystis* sp.	Cyclohexanone monooxygenase from *Acinetobacter* sp.	Oxyfunctionalization	Cyclohexanone Cyclohexenone 2‐Methylcyclohexan‐1‐one 3‐Methylcyclohexan‐1‐one 4‐Methylcyclohexan‐1‐one Cyclopentanone Cyclopentenone 5‐Isopropenyl‐2‐methylcyclohexanone	1.5	150	1.8	Glass vials	n.r. n.r. n.r. n.r. n.r. 70 n.r. n.r.	n.r. n.r. n.r. n.r. n.r. n.r. n.r. n.r.	2.3 n.c. 2.0 2.9 5.7 2.3 n.c. n.c.	[63]
6	*Synechocystis* sp.	Alkane monooxygenase from *P. putida* GPo1	Terminal hydroxylation	Methyl nonanoate	1	30	2	Glass tubes	0.65	1.04 × 10^−6^	1.5 0.9^b^	[54]
7	*Synechocystis* sp.	BVMO from *B. xenovorans*	C–H Oxyfunctionalization	Cyclohexanone	1	300	2.4	Glass vials	99	3.24 × 10^−5^	25.7	[36]
8	*Synechocystis* sp.	CYP450 monooxygenase CYP110D1	Hydroxylation	Testosterone	2	150	0.9–1.8	Glass vials	67	2.75 × 10^−7^	1.03	[60]
9	*Synechocystis* sp. (ΔhoxYH mutant)	Ketoacid dehydrogenase from *Lactobacillus* sp.	α‐ketoacid reduction	Phenylpyruvic acid 4‐Methyl‐2‐oxovaleric acid	1	215	2.4–4.8	GC vials	46 53	n.r. n.r.	n.r. n.r.	[100]
10	*Synechococcus elongatus* PCC 7942	Alcohol dehydrogenase	Ketone reduction	Acetophenone	n.r.	150	0.7	Shake flask	> 99	7.78 × 10^−6^	24	[101]
11	*Synechocystis* sp.	Ene‐reductase YqjM	Ene‐reduction	2‐Methylmaleimide *N*‐methylmaleimide 2‐Methyl‐*N*‐Methylmaleimide 2‐Cyclohexen‐1‐one	1.2	200	0.6–2.4	Glass vials	*ca.* 80 *ca.* 10 *ca.* 50 *ca.* 70	3.53 × 10^−5^ 1.77 × 10^−5^ 7.87 × 10^−5^ 1.32 × 10^−4^	150 30 135 150	[102]
12	*Synechocystis* sp.	BVMO from *Acidovorax* sp. CHX100	Oxyfunctionalization	Cyclohexanone	1	150	0.7–1.0	Pyrex tubes	n.r.	n.r.	60	[37]
13	*Synechocystis* sp.	Ketoreductase LfSDR1M50 Ene reductase YqjM Baeyer‐Villiger Monooxygenase CHMO_mut_	Reduction Ene‐reduction Oxyfunctionalization	4‐Methylcyclohexanone 2‐Cyclohexen‐1‐one Cyclohexanone	n.r.	n.r.	OD10^d^	n.r.	70* >99 60	n.r.	35.8 54.6 7.3	[97]
14	*Synechocystis* sp.	Flavin monooxygenase from *Methylophaga aminisulfidivorans*	Oxygenation	Indole	5	30–150	OD4^d^	Corning tubes/flasks	86	1.56 × 10^−3^	0.98	[103]
15	*C. reinhardtii*	Cyclohexanone monooxygenase from *Acinetobacter* sp. Native [Fe–Fe]‐hydrogenase	Oxyfunctionalization Hydrogen production	Cyclohexanone ‐‐	n.r.	26^c^	100 µg_*Chla*_ mL^−1^	Flasks	*ca.* 73 n.a.	1.31 × 10^−6^ 2.48 × 10^−4^	3.6 n.r.	[50]
16	*Synechocystis* sp.	YqjM OYE2 OYE3 TsOYE_2M GluER	Ene‐reduction	2‐Cyclohexen‐1‐one 2‐Methyl‐cyclohexen‐1‐one 3‐Methyl‐cyclohexen‐1‐one 4‐Isopropylcyclohexenone 2‐Methylmaleimide 2‐Methyl‐*N*‐Methylmaleimide	1	150	2.4	Glass vials	>99.9 >95.2 13.4 68.2 >99.9 >99.9	n.r.	57.8 25.4 n.d. 107.7 154.7 138	[46]
17	*Synechocystis* sp.	BVMO and lactonase from *Acidovorax* sp.	Cascade: oxyfunctionalization + lactonization	Cyclohexanone	1	150	0.7–1.0	Pyrex tubes	n.r.	0.41^a^	63.1	[44]
**(b) Up‐scaling**												
18	*Synechocystis* sp.	YqjM	Ene‐reduction	2‐Methylmaleimide	200	200	0.48–2.4	Bubble column reactor	>99	3.27 × 10^−5^	25.7	[82]
19	*Synechocystis* sp.	YqjM	Ene‐reduction	2‐Methylmaleimide	200	200	2.4	Bubble column reactor	>99	n.r.	100	[102]
20	*Synechocystis* sp.	BVMO from *Acidovorax* sp. CHX100	Oxyfunctionalization	Cyclohexanone	2000	150–700	0.6–1.0	Stirred‐tank PhBR	n.r.	0.21	30	[37]
21	*Synechocystis* sp.	BVMO + lactonase from *Acidovorax* sp.	Cascade: Oxyfunctionalization + lactonization	Cyclohexanone	2000	700–900	0.7–1.0	Stirred tank PhBR	96	6.5 × 10^−2^	*ca.* 20	[44]
22	*Synechocystis* sp.	Alkane monooxygenase AlkBGT	Terminal oxyfunctionalization	Nonanoic acid methyl ester	3000	100	1.6	Stirred‐tank PhBR	n.r.	n.r.	5.6	[42]
23	*Synechocystis* sp.	Cytochrome P450 monooxygenase	Hydroxylation	Cyclohexane	1200	150	0.5–0.8	Stirred‐tank PhBR	n.r.	n.r.	39.2	[43]
24	*Synechocystis* sp.	OYE3 YqjM TsOYE_2M	Ene‐reduction	2‐Methylmaleimide	120	300	3.6	Flat panel PhBR	>99	1.0	56.1	[46]

a Calculated from initial rate of product formation.

b Anaerobic conditions.

c During hydrogen production, low light intensity (2–6 µmol photons m^−2^ s^−1^) was used in the initial phases.

d No correlation between the cell density and dry weight were given.

* Estimated based on figures.

Abbreviations: CSTR – continuously stirred tank reactor. PhBR – photobioreactor. n.r.‐not reported.

The abovementioned enzymes are not cofactor‐dependent, allowing in vitro reactions using CFE of cyanobacteria. Oxidoreductases, on the contrary, require a cofactor and a subsequent regeneration system in the form of either a two‐enzyme system or using cellular metabolism as discussed in Section 2. The gene of an ene‐reductase YqjM from *B. subtilis* was introduced into the genome of *Synechocystis* sp. (Table 3, Entry 1) by homologous recombination [34] under the control of the strong light‐inducible promoter P_*psbA2*_, and was utilized for the reduction of various alkenes. The reaction was performed in small‐scale (1.5 mL) with a light intensity of 150 µmol photons m^−2^ s^−1^. Conversions reached >99% particularly with 2‐methylmaleimide producing an optically pure compound (>99% ee). Inhibiting the flow of electrons from PSII by addition of 3‐(3,4‐dichlorophenyl)‐1,1‐dimethylure (DCMU) significantly reduced the product formation, suggesting that asymmetric reduction is light‐dependent. It should be noted that there is still appreciable product formation after treatment with DCMU and in the dark stemming from stored carbohydrates to regenerate NAD(P)H. A volumetric productivity of 1.1 g L^−1^ h^−1^ was obtained using an increased substrate amount of 100 mg.

In a following work, enzyme production was increased by promoter design using the same YqjM ene‐reductase, which consequently increased the specific activity by 1.3‐fold. By changing from P_*psbA2*_ to the stronger promoter P_*cpcB*_, a higher intracellular YqjM concentration was obtained [35]. This was also demonstrated by expressing genes of several imine reductases (IRED) in *Synechocystis* sp. to reduce various imines [98]. A high product formation of 6.3 mM h^−1^ with a specific activity of 22 U g_DCW_^−1^ for the reduction of 5‐methyl‐3,4‐dihydro‐2H‐pyrrole was observed when the genes of IRED‐A (from *Streptomyces* sp. GF2587) controlled by the P_*cpcB*_ promoter was used. Surprisingly, the specific activity did not decrease with increasing cell density, contrary to previous observations [35]. Furthermore, dark reactions supplied sufficient cofactors to sustain the reaction, resulting in specific activities comparable to those observed under light conditions. Hence, imine reduction mediated by recombinant cyanobacteria can be considered not light‐driven and storage compounds are sufficient to regenerate the cofactor.

Storage compounds in the cells allow regeneration of NAD(P)H during dark reactions in cyanobacteria as previously demonstrated [98]. Using a carbon‐rich compound such as glucose or glycerol, NADPH and NADH are regenerated through the pentose phosphate pathway and glycolysis, respectively [27]. In a recent study by Loprete et al. [103], the addition of glucose (5 mmol L^−1^) during the biotransformation of indole under dark conditions showed comparable product yields with the light‐driven reaction at the lowest tested light intensity (30 umol photons m^−2^ s^−1^) indicating that indeed cofactor regeneration in the dark can provide appreciable product formation. Mixotrophic conditions during biotransformation, that is, the addition of glucose to an active, light‐driven biotransformation mediated by *Synechocystis* sp. harboring the ene‐reductase YqjM led to an increase in specific activity [102]. This can be attributed to increased NAD(P)H availability in the presence of glucose as shown using light‐induced NAD(P)H fluorescence kinetics. However, mixotrophic cultivation—addition of glucose during cultivation, did not improve the intracellular YqjM activity which could be due to a different regulation of the P_*cpc*_ promoter during cultivation.

In a recent study, various genes of ene‐reductases were expressed in *Synechocystis* sp. and tested for the reduction of a panel of prochiral substrates [46]. The ene‐reductases were chosen from Class I–III which were better characterized as compared to Class IV‐VI. GluER from *Gluconobacter oxydans* represented class Ic, OYE 2 and 3 represented class II while YqjM as well as OYE from *Thermus scotoductus* (TsOYE) represented Class III. Apart from the common substrates used in ene‐reductions mediated by recombinant cyanobacteria, 4‐isopropylcyclohexanone and citral were tested. The former is of particular interest due to its potential as a polymer precursor while the latter is a precursor to produce the fragrance (–)‐menthol. Small scale reactions were performed at a volume of 1 mL with a light intensity of 150 µmol photons m^−2^ s^−1^. Interestingly, OYE3 and TsOYE displayed higher activity toward the maleimides tested as compared to the well‐known YqjM (Table 3, Entry 16). Furthermore, OYE3 showed complete reduction of 4‐isoproylcyclohexenone (10 mmol L^−1^) within 1 h with a specific activity of 107 U g_DCW_^−1^ with negligible ketoreduction. However, citral was poorly converted showing only 0.6 mmol L^−1^ (6% conversion) after 1 h coupled with discoloration of the cells after 24 h incubation, suggesting toxicity of the compound. The high activity of OYE3 could be attributed to its high concentration in the cells as shown via Western blot analysis. In contrast, the low concentration of GluER showing only a faint band in the Western blot could explain its moderate activity.

Another important reaction in organic synthesis is C–H oxyfunctionalization, which introduces oxygen atoms into hydrocarbons, enabling the functionalization of complex organic molecules [104]. Enzymes such as mono‐ and dioxygenases, including cytochrome P450s, play a key role in this process due to their high selectivity. However, the broader application of these enzymes remains limited by the challenge of supplying oxygen into the reaction, oftentimes requiring specialized reactors to regulate the pressure. Moreover, the poor solubility of oxygen in aqueous media results in a low oxygen transfer rate (OTR) [105]. One promising approach for oxyfunctionalization involves using recombinant photoautotrophic microorganisms, which can supply the required oxygen through water‐splitting during photosynthesis. In comparison, growing chemoheterotrophic *E. coli* respire oxygen at a rate of 100 U g_DCW_^−1^ competing with the reaction for the available oxygen in the medium [105].

The proof‐of‐concept for utilizing cyanobacteria for oxyfunctionalization was demonstrated by Böhmer et al. [63] by expressing the genes of a cyclohexanone monooxygenase from *Acinetobacter* sp. in *Synechocystis* sp. Reactions were performed in batch with a volume of 1.5 mL using a dry cell weight of 1.8 g L^−1^. Specific activities ranging from 2.0 to 5.7 U g_DCW_^−1^ for the oxidation of various ketones were reported. However, due to the presence of endogeneous ADHs in *Synechocystis* sp., ketoreduction, that is the formation of alcohol, as high as 50% was observed. The oxidation was also shown to be light‐dependent, as very low product formation was detected in dark reactions. Furthermore, addition of the PSII inhibitor, DCMU, also decreased the product formation. Similar to ene‐reduction, increasing the cell density above 1.8 g L^−1^ did not improve the initial product formation rate mostly attributed to self‐shading.

The rate in which photosynthetic oxygen can drive oxidation reactions was shown by Hoschek et al. [54] by expressing the genes of an alkane monooxygenase from *Pseudomonas putida* GPo1 (AlkBGT) in *Synechocystis* sp. The recombinant strain was utilized for the terminal oxidation of nonanoic acid methyl ester (NAME) to hydroxynonanoic acid methyl ester (H‐NAME) in small batch reactions. Anaerobic conditions were established by purging oxygen from the reaction medium and vessel. Compared to aerated systems, an almost 2‐fold decrease in specific activity (1.5 to 0.9 µmol min^−1^ g_DCW_^−1^) was observed in anaerobic reaction systems hinting on the contribution of external oxygen to the product formation. In the absence of the substrate, an O_2_ evolution rate of 3.7 µmol min^−1^ g_DCW_^−1^ was determined. This would indicate that nearly 25% of the photosynthetically generated O_2_, if we assume no photorespiration, are utilized for the reaction. The higher *K*_*M*_ of oxygenases with respect to O_2_ (10–60 µM) and the low effective O_2_ concentration inside the cell would suggest that oxygenases capture the photosynthetically generated O_2_ before diffusing outside the cell.

The relatively low specific activities of oxygenases compared to ene‐reductases in photoautotrophic microorganisms have prompted further research into enzyme discovery to enhance product formation rates. The BVMO gene from *Burkholderia xenovorans* [36] was expressed in *Synechocystis* sp. for the oxidation of cyclohexanone to ε‐caprolactone. Specific activities as high as 25 U g_DCW_^−1^ was reported which was 10‐fold higher as compared to the previously studied BVMO gene from *Acinetobacter* sp. in *Synechocystis* sp. [63] To further improve BVMO activity, Flvs in cyanobacteria are deleted to funnel the electrons to the enzymatic reactions, similar to what was previously performed in an ene‐reductase produced in *Synechocystis* sp. [35] Flavodiironprotens in cyanobacteria protect the cells from photo‐inhibition under fluctuating light conditions by reducing excess oxygen to water creating a Mehler‐like water‐to‐water cycle [106]. Deletion of Flv1 led to a higher specific activity as compared to the wild type strain. Furthermore, expression of another BVMO from *Parvibaculum lavamentivorans* also showed increased product formation rates and specific activities in the oxidation of cylohexanone, demonstrating the robustness of the deletion mutant [36].

In another study, a BVMO from *Acidovorax* sp. CHX100 [37] was produced in *Synechocystis* sp. and similarly utilized for the oxidation of cyclohexanone to ε‐caprolactone. The oxidation was also shown to be light‐dependent with an optimal light intensity of 150 µmol photons m^−2^ s^−1^ in small‐scale, exhibiting a high specific activity of 60 U g_DCW_^−1^. In a follow‐up study, an enzymatic cascade reaction was established by coexpression of a BVMO and lactonase genes from *Acidovorax* sp. to produce 6‐hydroxyhexanoic acid directly from cyclohexanone [44]. In small scale reactions (V = 1 mL) with a cell density of 0.7–1.0 g_DCW_ L^−1^, a specific activity of 63.1 U g_DCW_^−1^ was observed. The intermediate ε‐caprolactone was not detected in the reaction mixture. BVMO reaction was also found to be rate‐limiting since a specific lactonase activity of 2840 U g_DCW_^−1^ was observed using ε‐caprolactone as the starting material.

Building on this concept of leveraging photosynthetic hosts for oxidative biotransformations, CHMO genes from *Acinetobacter* sp. was also heterologously expressed in the microalga *C. reinhardtii* exploiting photosynthetically produced molecular oxygen and NADPH [50]. In addition, the reaction was coupled to hydrogen photoproduction using the native [Fe–Fe]‐hydrogenases under anaerobic and microoxic conditions. Based on toxicity tests, a concentration of 5 mmol L^−1^ cyclohexanone was utilized in the oxidation. However, a substantial portion of the substrate (85%) was converted to the side‐product, cyclohexanol, due to the native ADHs present. Addition of fomepizole and ethanol to inhibit the activity of the ADH was tested, and the supplementation of the latter improved the yields. The conversion was also optimal at low light intensity (26 µmol photons m^−2^ s^−1^) which is attributed to the establishment of a high NAD(P)H to ATP ratio in microalgal cells. Moreover, low light conditions would decrease CO_2_ fixation and subsequently biomass formation. The conversion was further improved by physically associating CHMO with photosynthetically derived NADPH. This was achieved by modifying a strain to introduce the chloroplast transit peptide of PSAD, a subunit of PSI complex, to CHMO.

The selective hydroxylation of steroids, particularly testosterone, mediated by a heterologous cytochrome P450 (CYP450) expressed in *Synechocystis* sp. was demonstrated by Mascia et al. [60] Among redox enzymes, CYP450s find interesting applications in pharmaceuticals as well as in the synthesis of fine or bulk chemicals, hydroxylating non‐activated C–H groups using molecular oxygen as cosubstrate and two electron carriers (i.e., NAD(P)H‐dependent ferredoxin reductase and ferredoxin) under mild conditions and in a regio‐ and stereo‐selective way. By hydroxylating testosterone, its oral bioavailability will be improved with minimum impact on the liver. Moreover, the consistent therapeutic impact of hydroxytestosterone delivered at low dosages is believed to be related to the efficiency of its uptake [107]. The gene encoding CYP110D1 was expressed under the control of P_*trc.x.tetO2*_ promoter showing *ca.* 2‐fold higher level of proteins as compared to the P_*psbA2*_ promoter. The hydroxylation was light‐dependent, albeit showing minor conversions (16%) under dark conditions attributed to storage compounds such as glycogen or polyhydroxybutyrate. Similar with other biotransformations mediated by cyanobacteria, increasing the cell density to an optimum of 2.4 g_DCW_ L^−1^ (OD_750_ = 10) showed maximum product formation. Aeration and inorganic carbon supplementation in the form of NaHCO_3_ (50 mmol L^−1^) also boosted the conversion from 57% to 83%. In summary, a specific activity of 1 U g_DCW_^−1^ was calculated in the hydroxylation of testosterone. This is *ca.* 2‐fold higher than what was obtained in *E. coli* using glucose as sacrificial electron donor.

In a recent study, recombinant *Synechocystis* sp. was utilized as a host to produce indigo, a widely consumed vat dye in the textile industry, by expressing the genes of the flavin‐monooxygenase from *Methylophaga aminisulfidivorans* (*m*FMO) [103]. As mentioned previously, reaction conditions such as cell density, light intensity, and extraction media were optimized to successfully produce 112 mg L^−1^ of indigo with 86% conversion. An initial concentration of 1 mmol L^−1^ was shown to be optimal, and a further increase in concentration did not improve the product formation, which could be attributed to the compound's toxicity. Various light intensities were tested to optimize product formation. The highest conversion (85%) was achieved at the highest light intensity tested of 150 µmol photons m^−2^ s^−1^ signifying the light‐dependence of the reaction to produce the required cofactors. Downstream proce of indigo by extraction with various detergents as shown in *E. coli* proved detrimental to *Synechocystis* sp. showing decreased yields. Hence, physical adsorption of indigo on polymer materials were tested with polyamide‐netted films showing the highest product recovery. Indigo was then reduced using a mixture of NaOH and Na_2_S_2_O_4_ and the nets further reused for new cycles.

In summary, the influence of light intensity was evident in small‐scale reactions, for both ene‐reduction and oxidation, where higher light intensities resulted in increased yields and specific activities. Furthermore, mixotrophic conditions in light‐driven biotransformations enhanced cofactor availability, thereby improving overall productivity. In the following section, the upscaling of several of the aforementioned reactions is presented, and the corresponding effects on catalytic activity is discussed.

##### 3.1.2.2 Upscaling

Efficient light availability and utilization remain key challenges in upscaling light‐driven biotransformations. As mentioned in Section 3, light absorption by cells leads to the formation of light gradients within the reactor, where cells near the periphery of the reactor are effectively illuminated, creating the “self‐shading” phenomenon. This result is compounded at higher cell densities as well as increased diameter of the reaction vessel [79].

Internal illumination was evaluated to alleviate self‐shading in ene‐reductions mediated by *Synechocystis* sp. harboring the ene‐reductase YqjM from *B. subtilis* [82, 102]. The reaction was performed using a bubble column reactor (BCR) equipped with freely suspended wireless light emitters (WLEs) with a reaction volume of 200 mL. This concept was developed by Buchholz and coworkers for the large‐scale photoautotrophic growth of the microalga *C. reinhardtii* [108]. The WLEs contain a light‐emitting diode (LED) and a receiving coil enclosed in polymeric shell and could be mobilized by gas flow [109]. Hobisch et al. [82] showed that even at a high cell density of 2.4 g_DCW_ L^−1^, a moderate light intensity of 60 μmol photons m^−2^ s^−1^ can be delivered to the cells. Using the recombinant cyanobacteria expressing the genes of the ene reductase YqjM, the reduction of 2‐methylmaleimide was investigated. In comparison with external illumination provided by LED strips wrapped around the reactor, the activity decreased by around 81% at a cell density of 2.4 g_DCW_ L^−1^ compared to internal illumination. Thus, showing the effectivity of internal illumination in highly dense cultures. In another study using the same recombinant cyanobacteria and BCR with suspended WLEs, a 2.4‐fold increase in specific activity was observed when the reaction was supplemented with d‐Glucose. Apart from cyanobacterial biotransformations, the use of WLEs was also proven efficient in the photodecarboxylation of fatty acids to produce biofuels [109]. The high SVR of internally illuminated photobioreactors attributed to the various kinds of light emitting elements is their main advantage compared to externally illuminated ones.

Reactor diameter in photobioreactors often correlates to the effective optical path length. In this regard, a flat panel photobioreactor (FPBR) was employed to upscale an ene‐reduction mediated by *Synechocystis* sp. harboring the ene‐reductases YqjM, OYE3, and TsOYE previously mentioned in small‐scale batch reactions [46]. The FPBR features a reactor thickness of 1 cm and a high SVR of 310 m^2^ m^−3^, which enables efficient light penetration. Illumination was provided by LED lamps positioned on both sides of the reactor, delivering approximately 300 µmol photons m^−2^ s^−1^ across the 1 cm optical path length. Biotransformations were performed using a working volume of 120 mL. Syn::P_*cpc*_OYE3 was found to be the most effective showing a specific activity of 56.1 U g_DCW_^−1^ closely followed by Syn::P_*cpc*_TsOYE_2M (51.1 U g_DCW_^−1^) and Syn::P_*cpc*_YqjM (46.2 U g_DCW_^−1^). High yields were observed (>99%) even at higher cell densities of 3.6 g_DCW_ L^−1^, achieving an STY of *ca.* 1 g L^−1^ h^−1^. To date, this is the highest STY achieved at biotransformations with volumes exceeding 100 mL.

Stirred‐tank photobioreactors are oftentimes utilized for upscaling light‐driven biotransformations with working volumes reaching up to three liters [37, 42, 44]. Earlier upscaling efforts were focused on hydroxylation and terminal oxyfunctionalization mediated by a CYP450 [43] and an alkane monooxygenase [42], respectively. To investigate the long‐term applicability of *Synechocystis* sp. harboring AlkBGT in the terminal hydroxylation of NAME, reactions were performed in a Labfors 5 Lx (Infors AG) with a working volume of three liters. A higher light intensity up to 250 µmol photons m^−2^ s^−1^ increased the oxygen evolution rates (*ca.* 0.7 U g_DCW_^−1^). However, the higher oxygen evolution rates did not significantly improve the specific hydroxylation rates, reaching a maximum of 3.0 U g_DCW_^−1^ at a light intensity of 30 µmol photons m^−2^ s^−1^. The toxicity of both the substrate and product may have constituted to the low biotransformation yield. Thus, a two‐liquid approach using either ethyl oleate or diisononyl phthalate (DINP) were used as cosolvent. These organic solvents were utilized previously in whole‐cell heterotrophic bacteria. Ethyl oleate was detrimental to the cell's growth while DINP with 25% v/v dissolved NAME had no harmful effects on cell growth. Using a total culture volume of 3 L and a cell density of 1.6 g_DCW_ L^−1^, a specific activity of 4.5 U g_DCW_^−1^ was achieved with a biomass‐specific product yield of 2.6 mmol g_DCW_^−1^.

Although recombinant cyanobacteria generate molecular oxygen through water splitting—oxygen that can sustain the target reaction, as demonstrated in small‐scale studies [54]—large scale oxyfunctionalization processes generally still necessitate external aeration. Notably, the supplementation of additional oxygen has been reported to further enhance the specific activity of the reaction. In the aforementioned study, compressed air was supplied at an aeration rate of 1.8 L min^−1^ (2.2 vvm).

Aeration was also provided in BVMO‐mediated oxyfunctionalization in stirred‐tank photobioreactors [37, 44] with rates up to 2 L min^−1^ during cultivation, which was decreased to 0.2 L min^−1^ during biotransformation. As compared to small‐scale batch reactions, the highest specific activity decreased from 60 to 30 U g_DCW_^−1^ mainly attributed to product (i.e., ε‐caprolactone) inhibition, which was described as non‐competitive with a K_i,_ε_‐Cl_ of 0.83 mmol L^−1^ [37]. The optimized conditions using high light intensity (700 μmol photons m^−2^ s^−1^) and high carbon loading (CO_2_, 20 mL min^−1^) delivered a final product titer of 11.5 mmol L^−1^ with a biocatalyst‐based yield of 1.3 gε_‐Cl + 6‐HA_ g_DCW_^−1^. A substrate‐feed regime led to a moderate increase in the final product titer (15.5 mmol L^−1^) coupled with low biocatalyst‐based yield of 0.4 gε_‐Cl + 6‐HA_ g_DCW_^−1^ due to the increased biomass concentration. In a follow‐up study, a BVMO and a lactonase from *Acidovorax* sp. were co‐expressed in *Synechocystis* sp. and utilized for the cascade reaction from cyclohexanone to the polymer precursor, 6‐hydroxyhexanoic acid (6‐HA) [44]. Based on prior findings that ε‐caprolactone significantly affects the stability of the process, introducing a lactonase for its subsequent conversion to 6‐HA effectively mitigated this constraint. Under an optimized substrate‐limited regime of 10 U g_DCW_^−1^, a 6‐HA concentration of 23.5 mmol L^−1^ was achieved after 48 h with a biocatalyst‐based yield of 3.71 g_6‐HA_ g_DCW_^−1^. In addition, an energy balance was created to determine the amount of energy withdrawn from photosynthesis. This included the amount of incident light, photosynthetic reductants (e.g., NADPH) and the electron demands for biomass formation and biotransformation reactions. A maximum quantum efficiency for product formation (ϕ_P_) of 0.070 and a light‐to‐product efficiency of 1.8% were achieved at high substrate loading. However, this was reached in the first hour of the experiment and was not sustained in comparison to a lower substrate loading, which also afforded lower ϕ_P_ (0.026) and 0.7% light‐to‐product efficiency.

However, the impact of aeration in photobioreactors may be negative, depending on the volatility of the reactants, potentially reducing product formation rates. For instance, in a hydroxylation reaction catalyzed by a CYP450 monooxygenase from *Acidovorax* sp. CHX100 produced in *Synechocystis* sp., conducted in a 3 liter‐stirred‐tank photobioreactor, a notable decline in biocatalyst activity was observed after 5 h [43]. Specifically, the activity was found to be two‐fold lower than that observed in shake‐flask reactions. This was attributed to the evaporation of the substrate according to the measured cyclohexane hydroxylation kinetics, together with an increased pH from degassing of carbon dioxide. To overcome this, hydroxylation was performed without aeration while maintaining the oxygen partial pressure at 100% using in situ O_2_ from cyanobacteria.

Upscaling of light‐driven biotransformations is oftentimes challenging, with several key factors to consider, such as appropriate reagents and solvents, health and safety considerations, isolation and purification of the desired product, and analytical methodology. Moreover, the yield and AE of the process should be considered, making sure that the process is efficient and environmentally‐friendly alternatives are chosen. For example, isolation and purification of the desired compound in small‐scale reactions require flash column chromatography and substantial volumes of solvents. During upscaling, these amounts are compounded, which makes purification using crystallization or distillation more cost‐effective [110]. In this regard, enzymatic reactions mediated by recombinant cyanobacteria oftentimes produce a pure compound provided that the reactant or the reaction proceeds to full conversion. Preparative‐scale ene‐reductions catalyzed by YqjM produced in *Synechocystis* sp. showed outstanding enantioselectivities (>99%) and the product, (*R*)‐2‐methylsuccinimide, could be isolated after solvent extraction without the need for purification [34, 82, 111]. This proved to be an advantage using recombinant cyanobacteria as hosts.

Table 4 shows several productivity parameters in an upscaled whole‐cell biotransformation utilizing *Synechocystis* sp. The highest product titer (1 g L^−1^ h^−1^) was shown in the reduction of maleimides catalyzed by ene reductases (i.e*.*, YqjM or OYE3) (Table 4, Entries 4 and 7). These were acquired by utilizing internal illumination coupled with mixotrophic conditions [102] or by utilizing a FPBR with a thickness of 1 cm [46]. Efficient substrate utilization was also shown in several works exhibiting *ca.* 100% product over substrate yields. However, the low product titers (Table 4, Entries 1,2,5,6) as well as product and biocatalyst yield (Table 4, Entries 1,2) during upscaling of oxyfunctionalization reactions point out the difficulties in oxygen supply in the reactor. Direct comparison of small‐ and upscaled reaction systems is quite challenging to undertake because light distribution, substrate (and product) transport, and energy input vary non‐linearly with reactor size as discussed in Section 3. This complicates direct comparison of reported activities and highlights the need for careful normalization and standardized reporting.

**TABLE 4:** Productivity parameters on upscaled biotransformation reactions mediated by recombinant *Synechocystis* sp.

Entry	Reaction and Enzyme	LI, µmol photons m^−2^ s^−1^	V, mL	Productivity Parameters			Ref.
				Titer, g L^−1^ h^−1^	Biocatalyst yield, g_product_ g_biomass_^−1^	Product yield, g_product_ g_substrate_^−1^	
1	Terminal oxyfunctionalization AlkB from *P. putida*	100	3000	0.04	0.72	9.50 × 10−3^a^	[42]
2	Hydroxylation CYP450 from *Acidovorax* sp. CHX100	150	3000	8.28 × 10^−2^	3.20 × 10^−3^	0.17	[43]
3	Ene‐reduction YqjM from *B. subtilis*	*ca.* 270	200	0.21	0.09	1.02	[82]
4	Ene‐reduction YqjM from *B. subtilis*	*ca.* 270	200	1.13	0.47	1.02	[102]
5	Oxyfunctionalization BVMO from *Acidovorax* sp.	700	2000	0.10^b^	1.30	0.88	[37]
6	Oxyfunctionalization + lactonization (BVMO and lactonase from *Acidorax* sp.)	900	2000	0.06	3.71	0.96	[44]
7	Ene‐reduction Various Old Yellow Enzymes	300	120	1.0	1.66	1.06	[46]

a Calculated from data extrapolated from figures.

b Sum of ε‐caprolactone + 6‐hydroxyhexanoic acid.

Abbreviation: LI‐ light intensity.

### 3.2 Continuous Reactors

Continuous flow biocatalysis, where chemical reactions take place in a continuously flowing stream, are gaining increasing attention in recent years. For example, miniaturized flow reactors allow reactions in controlled conditions with reduced environmental impact and improved heat and mass transfer [112, 113]. Additional advantages include: a) ease in upscaling using the “numbering up” approach [114]; b) large interfacial area [115]; c) simple DSP; d) possibility for automation and installation of in‐line sensors, and e) increased operational safety [80, 114].

In the case of photobiocatalysis, the large interfacial area or SVR afforded by meso‐ and microreactors is a huge benefit enabling uniform irradiation across the reactor. As an example, using the model reaction of 2‐methylmaleimide reduction catalyzed by the ene‐reductase YqjM produced in *Synechocystis* sp., Valotta et al. (2022) [111] was able to increase the STY to 14.4 g L^−1^ h^−1^ by using a coil reactor compared to batch reactions. The low internal diameter of the reactor (φ_i_ = 2 mm) provided uniform irradiation even at higher cell densities (3.6 g_DCW_ L^−1^) mainly attributed to its high SVR of 2000 m^2^ m^−3^. In comparison with batch reactions for both small and upscaled [82], the initial rate of product formation was increased 3‐fold with the coil reactor.

Using the abovementioned coil reactor, the rate in which in situ O_2_ from cyanobacteria can enhance an oxidation reaction was demonstrated using a BVMO produced in *Synechocystis* sp. [55] Oxidative biotransformations are one of the most important organic reactions in the synthesis of pharmaceutical ingredients and fine chemicals, exhibiting high selectivities as compared to chemical oxidants. However, due to the poor solubility of O_2_ in aqueous medium at atmospheric conditions (1 atm, 25°C), reaction rates are limited. Homogeneous oxygen generated via water splitting by photoautotrophic microorganisms could potentially alleviate the gas–liquid mass transfer limitation in oxygen‐dependent whole cell biocatalysis. Oxygen sensors positioned at the outlet of the coil reactor recorded an increase in dissolved O_2_ concentration, reaching up to 700 µmoL L^−1^ when cyanobacterial cells were in contact with the sensor. *Synechocystis* sp. expressing the BVMO_*Xeno*_ genes as previously described [36] was utilized in the coil reactor to catalyze the oxidation of cyclohexanone to the polymer precursor, ε‐caprolactone. Similar with ene‐reduction in the coil [111], oxidation can also be performed at higher cell densities (i.e., OD15 corresponding to 3.6 g_DCW_ L^−1^) without drastically decreasing the specific activity. Comparing with heterotrophic *E. coli* harboring the same enzyme, a 10‐fold lower specific activity was observed when reactions were performed in the coil reactor without additional aeration as compared to a batch reaction with sufficient shaking (hence, aeration). This is attributed to respiration, with *E. coli* showing at least 60 U g_DCW_^−1^, competing with the reaction [105]. In contrast, there was no significant difference in specific activities when the reaction was performed using recombinant cyanobacteria demonstrating the advantage of utilizing O_2_‐generating photoautotrophic microorganisms in oxidation reactions, particularly in O_2_‐limited systems. A higher STY was also calculated in flow (2.8 g L^−1^ h^−1^) as compared to batch (0.4 g L^−1^ h^−1^) underscoring the effectiveness of performing the reaction in flow.

In another work leveraging the O_2_‐producing ability of cyanobacteria in continuous flow capillary reactor, a mixed specie system consisting of a heterotrophic biofilm‐former, *Pseudomonas taiwanensis* VLB120, and the O_2_‐producing *Synechocystis* sp. was established [116]. Both species expressed the cyclohexane monooxygenase genes for the oxidation of cyclohexane to cyclohexanol. Proto‐cooperation was used between the species to achieve high cell densities of up to 51.8 g_BDW_ L^−1^ (biomass dry weight). In addition, the low internal diameter (0.5–3 mm) of capillary bioreactors (CBRs) allows maximal utilization of light energy due to its high SVR (1333–4000 m^2^ m^−3^) [117]. A productivity of 0.16 g m^−2^ h^−1^ and conversions as high as 98% were reported. However, the low initial substrate concentration (1 mmol L^−1^) and long duration (37 days) of film formation represent a limitation of this promising system. Albeit not considered biotransformation, a similar concept was adapted in the whole‐cell production of hydrogen using filamentous and diazotrophic cyanobacteria, *Nostoc punctiforme* and *Anabaena* sp. PCC 7120, lacking H_2_ uptake hydrogenases [118]. A final biocatalyst titer of 100 g L^−1^ was achieved with H_2_ production rates up to 300 µmol H_2_ L^−1^ h^−1^ and 3 µmol H_2_ g_DCW_^−1^ h^−1^ in the biofilms.

Investigations into whole‐cell photoautotrophic biotransformations operated in continuous flow reactors are comparatively sparse. The following examples described flow biotransformations performed in vitro with purified cells due to the instability of the photoenzymes in whole cells. In particular, the fatty acid photodecarboxylase from *Chlorella variabilis* (*Cv*FAP) that has been used to produce biofuels in batch reactors [109, 119] has also been used in flow [120]. Depending on the photo‐lability of the catalyst, process parameters such as light intensity should be optimized. In the case of *Cv*FAP, a lower light intensity of 42 µmol photons m^−2^ s^−1^ showed higher conversions as compared to a 10‐fold higher light intensity (455 nm) which could be attributed to the photodegradation of the photocatalyst. The flow set‐up consists of a packed‐bed reactor requiring immobilization of *Cv*FAP on various commercial resin supports. The highest immobilization yield and consequently, highest conversion achieved was using the purified enzyme in the EziG Opal resin. The continuous process was able to achieve an STY of 5.7 g L^−1^ h^−1^ in the photo‐decarboxylation of palmitic acid to pentadecane [120].

Using the same enzyme, a microfluidic reactor was developed for the continuous decarboxylation of palmitic acid using *Cv*FAP [121]. Microfludic reactors offer key advantages over batch reactors such as improved mass transfer, easier industrial scale‐up, and, specifically for photobioreactors, reduced light attenuation. The microfluidic reactor utilized in the study has an inner diameter of 500 µm with a total reaction volume of 350 µL. Under optimized conditions, a 97% conversion was reached with a pentadecane formation rate of 60 mmol L^−1^ h^−1^ (13 g L^−1^ h^−1^), and a turnover frequency of 19,186 h^−1^.

## 4 Immobilized Systems

Another requirement for industrial application of a biocatalyst is its stability, which can be improved by immobilization. Techniques such as entrapment, encapsulation, adhesion, covalent bonding, or a mix of these are frequently applied to the biocatalyst. To tap into the pool of reducing equivalents in photoautotrophic microorganisms, whole‐cells are immobilized offering an effective, ecological, and low‐cost catalyst [122]. Other advantages of immobilization apart from increased stability include the ease of separating the catalyst from the reaction mixture. This, in turn, simplifies DSP (e.g., product separation and purification) and increases the total turnover number (moles of product/moles of catalyst) of the catalyst through reuse [123, 124].

In particular, photosynthetic cell factories (PCFs) are immobilized as films to increase cell density allowing improved light utilization on a per area basis [125]. This would prevent “self‐shading” even at higher cell loadings, mostly encountered in batch reactions.

### 4.1 Cell Entrapment Using Various Polymers

**FIGURE 6:**
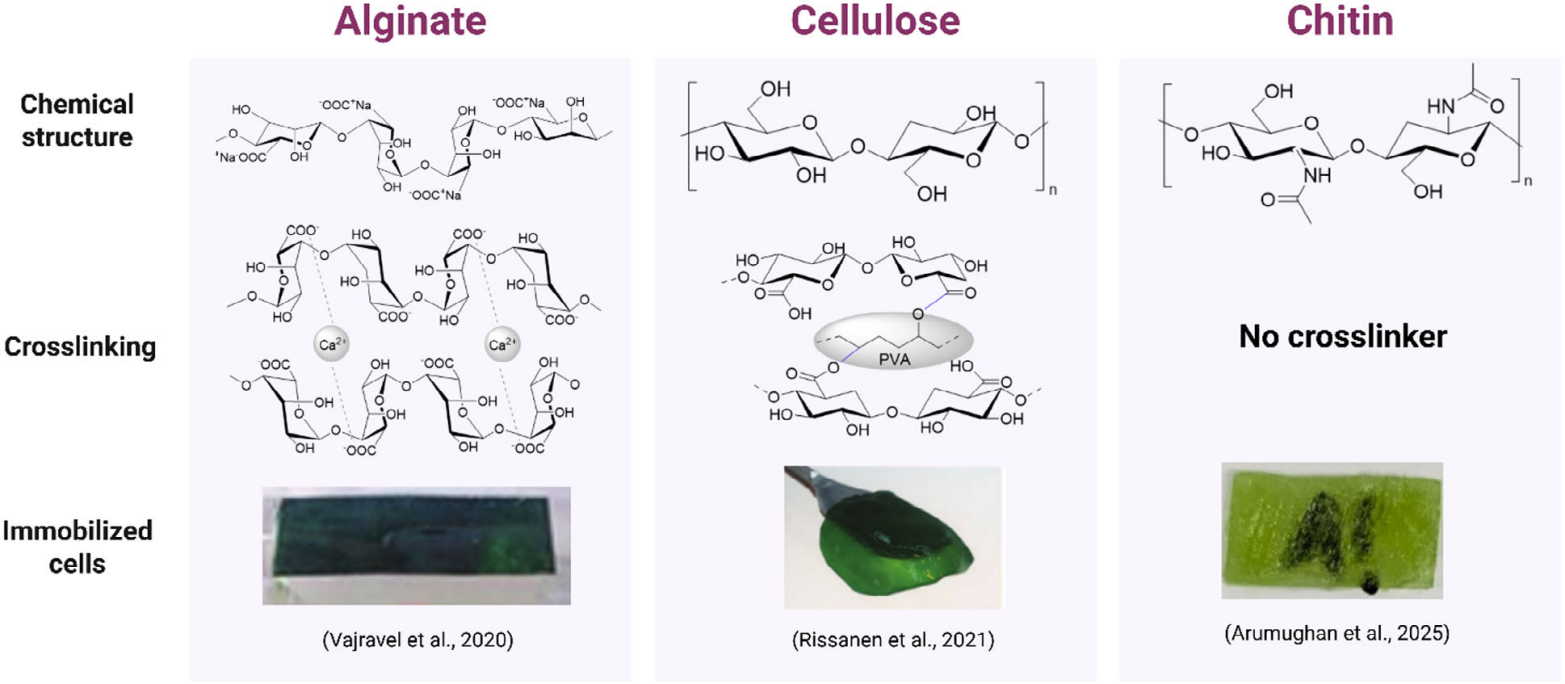
Polymers employed to immobilize photoautotrophic microorganisms for whole‐cell biotransformations. Alginate is the most commonly employed bio‐polymer to entrap photoautotrophic microorganisms due to its biocompatibility with living cells [126]. TCNF‐based films are self‐standing matrix architecture obtained from cellulose nanofibers operating in challenging submerged conditions [127]. Nanochitin‐based matrices deliver optimized light distribution owing to its microporous nature [128]. The images of immobilized cells were reproduced from references [126, 127, 128].

Whole‐cells of photoautotrophic microorganisms are oftentimes immobilized via entrapment using natural polysaccharides such as alginate, chitosan and agar–agar [125]. The polymer must be robust enough to entrap the cells, yet sufficiently porous to allow diffusion of substrates and products. Figure 6 shows various polymers used in the entrapment of photoautotrophic microorganisms with their corresponding chemical structure and type of cross‐linking.

#### 4.1.1 Alginate

Alginate has been exhaustively used to entrap microalgae [125, 126, 129]. The polymer consists of 1‐4‐linked‐ß‐D‐mannuronic acid and α‐L‐guluronic acid arranged in varying proportions and sequences and is commercially derived from brown algae. One of its major advantages is that alginate gel entrapment protects cells from drastic physical or chemical changes during the immobilization process [130]. In addition, 4‐6% (w/v) alginate solutions typically exhibit a pH close to 7, which is favorable for cell viability [125]. Cross‐linking with non‐toxic divalent ions such as Ca^2+^ and Ba^2+^ (Figure 6) initiates gel formation and can be easily carried out at room temperature.

In a previous review [21], we have already introduced immobilization of photoautotrophic microorganisms as thin films to increase its light utilization efficiency. Briefly, Kosourov and Seibert (2009) [125] have established a method for preparing thin alginate films using a solution of sodium alginate and microalgal cells (*C. reinhardtii*). In Figure 6, alginate‐based materials require a support material to maintain its structural integrity. In the aforementioned study, an insect screen was utilized to hold the thin film, which was formed after spraying with a solution of CaCl_2_ (50 mmol L^−1^). The films had an average thickness of *ca.* 300 µm with a volume of 200 µL for a 6 cm^2^ strip. By immobilizing as thin films, the cell density was increased 130‐fold times as compared to suspension culture. The films were then submerged in anaerobic TA‐S‐P medium, producing a maximum of 0.30 mol m^−2^ of H_2_ after 140 h. This concept of immobilizing photoautotrophic cells in alginate was also demonstrated in the production of ethylene (C_2_H_4_) mediated by *Synechocystis* sp. harboring the ethylene forming enzyme (*efe*) [126]. A foam sponge was used to support the thin films showing continuous ethylene production for 144 h. An optimum substrate concentration of 200 mmol L^−1^ NaHCO_3_ was determined delivering 10.95 mmol m^−2^ C_2_H_4_. Extended production of ethylene was also observed in immobilized films (38 days) as compared to suspension reactions (15 days). Furthermore, the light‐to‐ethylene conversion was determined to be 3‐fold higher in immobilized cells (0.35%) compared to suspension reactions (0.10%) further affirming the benefits of immobilization.

Spherical geometries—beyond thin films—have also been developed using alginate‐cyanobacterial mixtures for use in biotransformation processes. Early studies reported the immobilization of wild‐type unialgal strains in sodium alginate beads for the biotransformation of monoterpenes [131]. Encapsulating whole‐cells in alginate improved long‐term stability, facilitated product recovery, and enabled cell reuse. Among the monoterpenes tested, ß‐pinene was not converted by all the strains, whereas most strains were able to transform carvone and limonene.

Using genetically‐engineered *Synechocystis* S02, known to secrete sucrose up to 1800 mg L^−1^, a coupled microbial production system with recombinant *E. coli* co‐expressing the genes of an invertase and a BVMO was established [132]. Various strains of cyanobacteria are known to synthesize sucrose to alleviate osmotic pressure in the presence of salts through an inherent sucrose pathway [133]. By heterologously expressing a sucrose permease, encoded as *cscB* from *E. coli*, strains such as *Synechococcus elongatus* sp. PCC 7942, UTEX 2973 and *Synechocystis* sp. are able to secrete sucrose into the medium. Inactivation of the glucosylglycerolphosphate synthase (GGPS) as well as overexpression of the sucrose phosphate synthase (SPS) boosted the production of sucrose by *Synechocystis* sp. S02 [134]. In the study by Tóth et al. [132], the cyanobacterial cells were formulated into alginate beads and coupled to the aforementioned recombinant *E. coli* to produce the polymer precursor, ε‐caprolactone. Photosynthetically produced sucrose was then broken down to its monomers, glucose and fructose, to boost the regeneration of NADPH via the glycolytic pentose phosphate pathway. The system was considered semi‐continuous since the immobilized cells are allowed to secrete sucrose first, which in turn, is then fed to the recombinant *E. coli* with the addition of cyclohexanone to produce ε‐caprolactone. Over a 7‐day period, a sucrose concentration of 1,150 mg L^−1^ was produced by *Synechocystis* S02. Full conversion of cyclohexanone (5 mmol L^−1^) was observed after 3 h following supplementation of photosynthetically derived sucrose with a product formation rate of 0.9 mmol L^−1^ h^−1^.

In a follow‐up work, the rate of product formation was increased by utilizing a “faster” BVMO from *B. xenovorans* [135] previously described [36]. The expression of the invertase gene either in the periplasm or the cytoplasm was also studied with the former showing higher conversion. Using photosynthetically derived sucrose, a product formation rate of 1.7 mmol L^−1^ h^−1^ was observed, which was *ca.* 2‐fold higher as compared to previous study [132].

Strengthening crosslinking in films often involves combining polymers with ionic interactions, such as using polyvinyl alcohol (PVA) and Ca^2+^ to achieve mechanical stability. However, these approaches typically require drying steps that impose stress on embedded cells and can lead to uneven, heterogeneous films. Osmotic dehydration offers a way to form more homogeneous films with controlled pore size and water content, but it is time‐consuming and makes it difficult to precisely regulate film thickness [136].

A new approach to tackle these challenges is to implement 3D‐printing using a novel bio‐ink comprising of photocurable materials, alginate and photosynthetic microbes to enhance the mechanical stability, and be able to design structures to improve mass transfer [137]. Cells of either *Synechocystis* sp. or the microalga *C. reinhardtii* were incorporated into a mixture containing alginate, galactoglucomannan‐methacrylate (GMMA) and a photo‐initiator, lithium phenyl‐2,4,6‐trimethylbenzoylphosphinate (LAP) to create thin films via 3D‐printing and photocuring. The recombinant cyanobacterium harbors *efe*, while *C. reinhardtii* harbors the BVMO enzyme, CHMO. The concentration of the photoinitiator was initially assessed to determine its compatibility with whole cells. A concentration of 0.05% LAP was deemed effective without showing any detrimental effects to cell growth. The thin films produced from the formulation exhibited higher levels of elasticity and shear stress tolerance as compared with Ca^2+^‐Alg or dried Ca^2+^‐PVA‐TCNF. Entrapped *C. reinhardtii* expressing the BVMO gene was able to produce 87% of ε‐caprolactone from the oxidation of cyclohexanone while *Synechocystis* sp. producing *efe* was able to produce 780 µmol mg_*Chla*_^−1^ ethylene with a specific formation rate of 7.3 µmol mg_Chla_^−1^ h^−1^. The higher mechanical stability of the 3D‐printed films was demonstrated when it showed higher rigidity in the presence of 200 mM sodium bicarbonate as compared to films entrapped in sodium alginate. Moreover, continuous production of ethylene was sustained for 20 days showing the stability of the immobilized system.

In most examples, reactions were performed in batch with a maximum of 10 mL of reaction volume. A lower light intensity (close to 30 µmol photons m^−2^ s^−1^) was also deemed optimal for the thin film production systems as compared to suspension due to their high light to energy efficiency [126, 138].

#### 4.1.2 Nanocellulose‐Based

As mentioned previously, alginate‐based matrices are commonly used for the entrapment of photosynthetic microorganisms. However, they perform poorly in environments with high ionic strength—such as wastewater, disrupting their ionic linkages [127]. In the context of ethylene production, the high optimal concentration of the bicarbonate substrate (200 mmol L^−1^ HCO_3_^−^) further complicates the process, necessitating the use of support materials to prevent Ca^2+^ precipitation, disrupting the matrix.

To increase the mechanical stability of thin films, a novel solid‐state cell factory matrix was designed to efficiently entrap cyanobacteria toward bio‐ethylene production. The films do not require a support material and are termed “self‐standing” made from never‐dried hydrogel thin films from TEMPO‐oxidized cellulose nanofibers (TCNF) [127]. Treatment of cellulose with 2,2,6,6‐tetramethylpiperidine‐1‐oxyl radical (TEMPO) selectively converts almost all C6 primary hydroxyl groups to sodium carboxylate groups preserving the crystallinity and microfibril dimensions. The resulting gels are transparent after mild agitation using a blender‐type or ultrasonic homogenizer [139]. The transparency can also be attributed to its small fibril diameter and high aspect ratio allowing efficient light penetration. Furthermore, its high porosity together with high surface area makes them suitable for light‐driven biotransformations [138]. Levä et al. [140] has demonstrated that Ca^2+^linked TCNF showed the highest mean pore size (52 nm) among various formulations including alginate crosslinked with Ca^2+^. Apart from calcium ions, interfibrillar bridging can be enhanced by using polyvinyl alcohol (PVA) (Figure 6).

TCNF‐based PCFs were initially utilized in the production of hydrogen via thin films following three formulations [138]. Support materials such as paper or insect screen were either used as foundation or incorporated in the matrix to increase the mechanical stability, respectively. A drying stage (70% relative humidity, 23°C, 22 h) was found to produce films with increased mechanical stability and wet strength. However, the drying stage significantly affected the photosynthetic activity of cells, with the quantum yield (QY or Y(II)) dropping to a value of 0.1 from 0.5. Cell recovery was achieved by rewetting for 4 days, which restored the Y(II) values to approximately 0.4 –an acceptable level for photosynthetic microorganisms [141]. However, H_2_ production remained higher in cells immobilized using only Ca^2+^‐alginate [138].

Albeit including a drying stage to increase the mechanical stability of the films, a support material in the form of melamine foam was still utilized during the reaction. In a succeeding work, TCNF films were utilized in the production of ethylene using *Synechocystis* sp. producing the ethylene forming enzyme from *Pseudomonas syringae* [127]. In contrast with the previous work [138], cells maintained good photosynthetic activity with Y(II) values ranging from 0.35 to 0.40 after drying. This recovery was observed only in films retaining 20‐70% water, whereas films containing 10% water or those fully dried did not regain functionality after rehydration. The resulting thin films were “self‐standing” and were used in the reaction under submerged conditions. Using the optimal substrate concentration of HCO_3_^−^ (200 mmol L^−1^) from Vajravel et al. [126], films maintained their rigidity for 8 days producing *ca.* 180 µmol C_2_H_4_ mg_Chla_^−1^. In contrast, control reactions using thin films composed of 4% alginate crosslinked with Ca^2+^ were unable to withstand the high substrate concentration and deteriorated within 20 min of NaHCO_3_ addition affirming the higher mechanical stability of TCNF‐based films [127].

In a recent study, Kosourov et al. [83] utilized TEMPO‐oxidized cellulose nanofibers to create a highly efficient biocatalytic architecture in the production of H_2_ using wild‐type microalga *C. reinhardtii*. This was done by systematically optimizing culture selection and light distribution through an antenna‐mutant gradient together with product removal using a semi‐wet configuration. *C. reinhardtii* mutant cells, having truncated antenna (Tla2) were placed on top of wild‐type cells having normal antenna sizes in a sandwich‐like architecture. This approach would optimize light distribution, minimizing energy loss associated with non‐photochemical quenching. The engineered photosynthetic biocatalysts produced four times more hydrogen than conventional alginate films under identical light conditions (0.65 vs. 0.16 mol m^−2^) and sustained activity for over 16 days. Peak light‐to‐hydrogen conversion efficiency reached 4%, remaining above 2% throughout the production period.

#### 4.1.3 Nanochitin

Porosity of the matrix significantly affects the product formation rates due to enhanced mass transfer. In a recent study, a new immobilization matrix was developed through freeze‐drying of nanochitin building blocks [128]. Nanochitins, composed of fibrillar *N*‐acetylglucosamine polymers, can be partially de‐acetylated to introduce surface amine groups that enable fiber disassembly and versatile crosslinking [142]. In contrast to alginate and TCNF‐based matrices, entrapment of cells in nanochitin does not require a crosslinker (Figure 6). A suspension of cells (1 mL) was drop‐casted into chitin hydrogels (1 x 3 cm), which was then dried to remove *ca.* 20% of water content. The loosely bound cells were removed after mild agitation for 1 h and further rinsed with BG‐11 medium prior use. Among the formulations tested, the medium‐length nanochitin (M‐ChNC) showed the best properties in terms of light transmittance (78%) and porosity (>95%). Scanning electron microscopy (SEM) images also revealed that the nanochitin‐based matrices exhibit a microporous structure, representing a significant enhancement compared with the submicron pores of alginate and TCNF. An average pore diameter of 150 µm was determined for M‐ChNC cryogels, which are *ca.* 3000‐fold higher than previously studied TCNF and alginate matrices [140]. During reduction of 2‐Methylmaleimide mediated by recombinant *Synechocystis* sp. harboring the ene‐reductase YqjM, a higher activity was observed in the cells immobilized in M‐ChNC as compared to suspension reactions. Furthermore, it showed the highest activity among other immobilization matrices such as alginate and TCNF, which could be attributed to its high porosity and light transmittance.

Reactions in immobilized systems may prove to be more sustainable than suspension reactions due to water savings during cell cultivation. As shown in abovementioned studies, several reaction cycles can be performed in immobilized cells, and the DSP is less complicated avoiding additional energy from separating the cells from the reaction mixture.

## 5 Overcoming Challenges in Whole‐Cell Photoautotrophic Biotransformations

### 5.1 Using ‘Faster’ Growing Strains

Another main bottleneck toward industrial applicability of photoautotrophic microorganisms as whole‐cell catalysts, beyond constraints from the allowable cell density during biotransformation, is their inherently slow growth and modest biomass accumulation. To address this, several “fast‐growing” cyanobacterial strains have been developed as alternatives to *Synechocystis* sp. for biotechnological applications.

The most widely used cyanobacterium, *Synechocystis* sp. exhibits a growth rate of 1.32 days^−1^ at a photon flux of 150 µmol photons m^−2^ s^−1^ with 5% CO_2_ [143], and a doubling time of 5–6 h under CO_2_‐enriched conditions [144, 145]. In 2015, the first reported fast‐growing strain *Synechococcus elongatus* UTEX 2973, demonstrated a remarkably short doubling time of 1.9 h in BG‐11 at 41°C, 500 µmol photons m^−2^ s^−1^ and 3% CO_2_. Under comparable conditions, this doubling time is at least two‐fold faster as compared to *Synechococcus elongatus* PCC 7942 and PCC 7002.

Similar with *Synechocystis* sp., genetic toolboxes were also developed for *Synechococcus elongatus* UTEX 2973 including the introduction of Tfp pilus to regain its natural transformability, evaluation of various promoters and lactose induction systems [146]. The strain was previously engineered to utilize xylose (photomixotrophy) by introducing the xylose utilization pathway from *E. coli*, followed by a rewiring of the native glycolytic pathway to drive the carbon flux toward acetyl‐CoA [147]. The production of 3‐hydroxypropionic acid (3‐HP) was then demonstrated via heterologous introduction of a 3‐HP biosynthetic pathway in UTEX 2973. Under photomixotrophic conditions, a 14‐fold increase in 3‐HP production (91.3 mg L^−1^) was observed as compared to photoautotrophic conditions.

In another study on 3‐HP production, *S. elongatus* UTEX 2973 was co‐cultivated with recombinant *E. coli*, where the cyanobacterium secreted sucrose that the engineered *E. coli* subsequently converted into 3‐HP [148]. The sucrose permease‐coding gene, *cscB* was overexpressed in UTEX 2973 to efficiently excrete sucrose produced by cyanobacteria to relieve osmotic pressure. This was then utilized by *E. coli* with a sucrose catabolic and malonyl‐CoA‐dependent 3‐HB biosynthetic pathway introduced, producing 68.3 mg L^−1^ 3‐HP directly from CO_2_. The potential of UTEX 2973 for the biosynthesis of other compounds such as polyhydroxybutyrate was also reported [149] producing a maximum of 420 mg L^−1^ in 10 days under photoautotrophic conditions. Metabolic engineering efforts are also focused on the improvement of sucrose secretion toward increased acetyl CoA production.

*Synechococcus* sp. PCC 11 901, another fast‐growing strain, was first described in 2020 [150] as a novel marine cyanobacterial strain. Some of its attractive features for biotechnological applications include higher temperature tolerance (up to 43°C), high light intensity (660 µmol photons m^−2^ s^−1^) and tolerance of high salinity (<1.8% w/v), the latter is beneficial as it can utilize wastewater. Under optimal growth conditions of 38°C and 1% v/v CO_2_, it exhibited a doubling time of 2.14 h at the aforementioned high light intensity reaching a biomass yield of 33 g_DCW_ L^−1^ [150]. A genetic toolbox was also established for PCC 11901 [151]. However, not only recently, that the strain has been exploited as a host for whole‐cell biocatalysis.

By expressing the genes of the phenolic acid decarboxylase (PAD) from *Bacillus coagulans* to its genome, *Synechococcus* sp. PCC 11 901 was able to convert cinnamic acid derivatives into hydroxy‐styrenes [66]. In particular, ferulic acid, *p*‐coumaric acid and caffeic acid were efficiently converted (80%‐100% substrate consumption, 10 mmol L^−1^ starting concentration). Discoloration—indicating toxicity from both ferulic acid and other substrates—was also observed, which was primarily attributed to the toxicity of the product effecting oxygen inhibition. In situ product removal was employed to alleviate product toxicity by testing various solvents (e.g., ethyl acetate, limonene, cyclopentyl methyl ether (CPME), DINP, isopropyl myristate (IPM), etc.). DINP and IMP proved to be the most suitable extraction solvents without detrimental effects on cyanobacterial pigments, chlorophyll *a* and carotenoids, and was subsequently used to convert 80 mmol L^−1^ of ferulic acid (fed‐batch) after 50 h. Finally, using a CellDEG system to cultivate cells to high optical densities, a fed‐batch system was set‐up using 0.78 g of ferulic acid (added in two portions) to achieve 1.19 g of 4‐vinylguaiacol. Although decarboxylation reactions are performed, the overall process does not result in net CO_2_ fixation and therefore cannot be classified as carbon‐negative whole‐cell catalysis.

### 5.2 Shuttling of Photosynthetically Derived Electrons

The difficulty to heterologously produce enzymes as well as the transport of substrate or products across the cell membrane have prompted a study that developed modular photo‐electron shuttling (MPS) system using photosynthesis. Using this approach, cloning is avoided, enzyme and substrate toxicity issues are alleviated, and transport limitations are bypassed [152]. Moreover, as discussed in previous sections, since most cyanobacteria are polyploid, expressing genes in their genome would take long periods compounded by extensive selection of genetic elements such as promoters. These difficulties are circumvented by shuttling the electrons photo‐generated by cyanobacteria outside of the cell to drive any redox reaction of interest. Briefly, it encompasses three modules, including a shuttle module involving a small module (e.g., ketone) that can pass through the cell membrane, taking up one redox equivalent. It then gets re‐oxidized in the 2nd module by an external alcohol dehydrogenase, producing either NADH or NADPH depending on the ADH's cofactor specificity. Finally, the reduced cofactor is utilized by the target enzyme in the 3rd module. The concept was tested using ene‐reduction as the 3rd module with wild type *Synechocystis* sp. in the 1st module reducing a shuttle pair (acetone/2‐propanol). The alcohol, 2‐propanol, gets re‐oxidized again via an NADPH‐dependent ADH from *Lactobacillus kefir* (*Lk*ADH) recombinantly produced in *Synechococcus elongatus* PCC 7942. Using the purified enzyme, conversions as high as > 99% can be achieved in the reduction of 4‐ketoisophorone with an enantioselectivity of 97%. The MPS was also extended to Baeyer‐Villiger oxidation, producing 84% of ε‐caprolactone from the oxidation of cyclohexanone.

Thus, the MPS system can alleviate some of the limitations of cyanobacterial biotransformations particularly those related to cofactor regeneration and mass transfer across the cell membrane. However, caution is still required when working with toxic substrates, when selecting ADHs that may undesirably react with these substrates, and during scale‐up, where self‐shading can reduce light penetration.

## 6 Sustainability

Previous sections have shown the broad diversity of options that photobiocatalysis may offer, producing different enzymes of interest for synthetic purposes. Aspects like AE and RME show promising environmental metrics, as better use of resources is achieved. However, for an in‐depth sustainability consideration, other aspects such as the total Global Warming Potential (GWP, kg_CO2_ kg_product_^−1^) of the established processes must be addressed [153]. In particular, comparisons of the newly established systems with industrially running biocatalytic processes should be made to illustrate where the potential (environmental) advantages can be found and hotspots improved.

From a broad perspective, the upstream of both systems would be conducted in aqueous solutions at mild temperatures, and the downstream would include an extractive step with an organic solvent, from which a number of bio‐based derivatives have been recently proposed [154]. In the upstream, two main contributors to GWP can be expected, namely the CO_2_ released within the wastewater treatment plant (WWTP) [155, 156], and the contribution of the energy needed for the reaction, which can be estimated from thermodynamic calculations [77, 157]. Adapted to the comparison between industrial biotransformations and photobiocatalytic systems, both equations can be drawn as

$$\text{GWP equation for WWTP}:\text{GWP}\left(\text{mild WWTP}\right)=\frac{0.073}{\left[P\right]}$$

$$\text{GWP equation for the energy needed in the upstream}:\mathrm{GWP}\left(\text{water energy}\right)=F\cdot \left[\left(\frac{0.00145\cdot \Delta T}{\left[P\right]}\right)+t\cdot \left(\frac{0.00022\cdot \Delta T}{\left[P\right]}\right)\right]$$

where [*P*] is product titers (in kg L^−1^; F is the factor associated to the CO_2_ production per kWh depending on the energy source (0.7 kg_CO2_ kW‐h^−1^ for fuel‐based processes; 0.25 kg_CO2_ kW‐h^−1^ for the European grid electricity, and 0.04 kg_CO2_ kW‐h^−1^ for renewable electricity (hydropower) [157]; ΔT is difference of temperature from the starting temperature (normally room temperature, 20°C) to the reaction temperature (in °C); and t is the reaction time (in h). Therefore, the total GWP of the upstream would be the sum of both contributions, WWTP impact and energy associated CO_2_ released.

**FIGURE 7:**
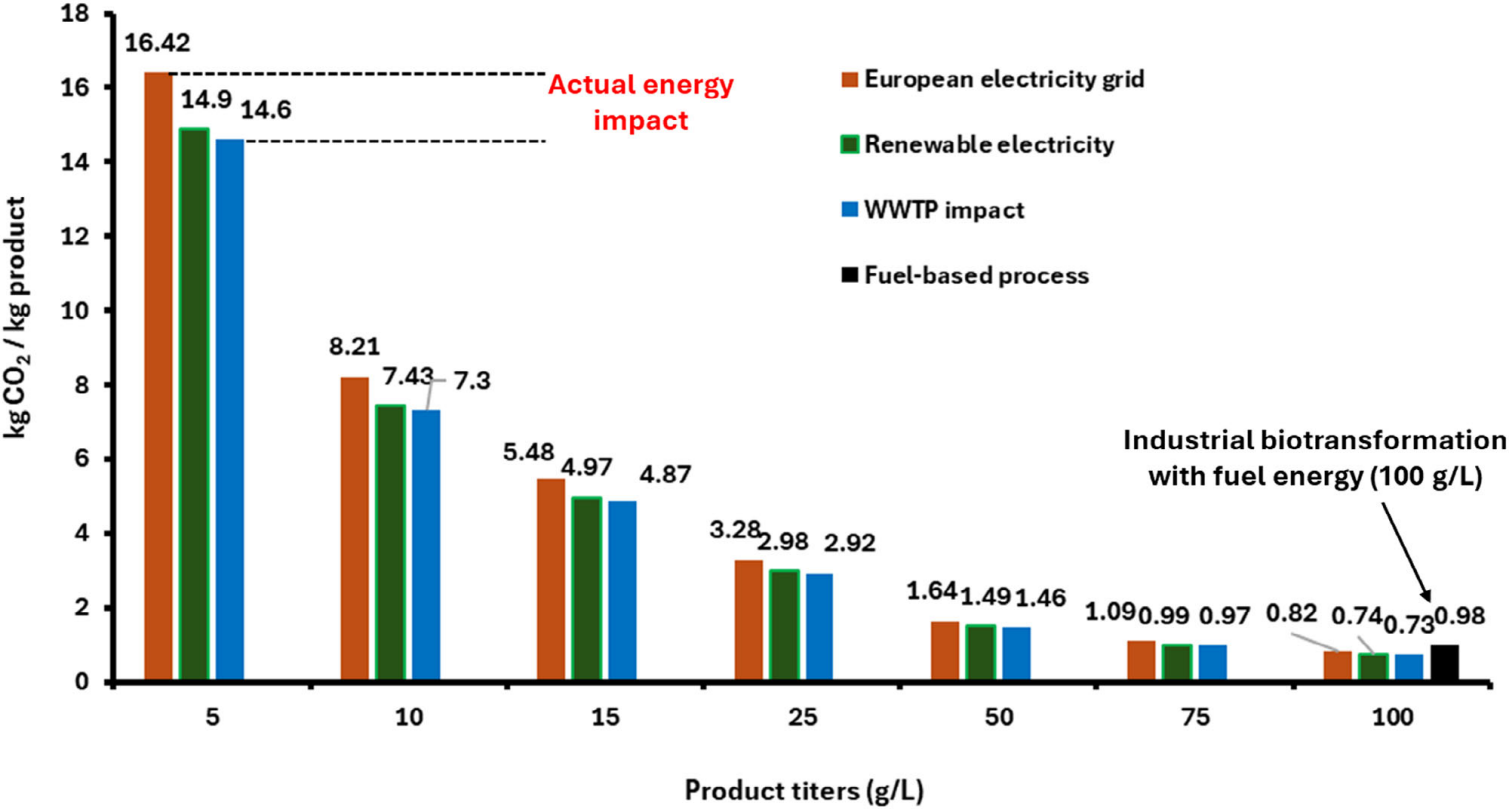
Estimated GWP (kg_CO2_ kg_product_^−1^) for the upstream part of photobiocatalytic reactions at several product titers, and different electricity sources, compared with a classic industrial biotransformation run with fuel‐based energy at 100 g L^−1^. WWTP‐wastewater treatment plant.

Herein, photobiocatalytic systems were environmentally compared with an established generic industrial biotransformation. Both processes were conducted in water at 30°C during 10 h. It is assumed that the generated wastewater could be mildly treated in the WWTP (0.073 kg_CO2_ L_wastewater_^−1^) [155]. For the industrial classic biotransformation, a product titer of 100 g L^−1^ was considered [158], and for the photobiocatalytic process, product titers from 5 to 100 g L^−1^ were simulated. To validate the influence of the energy source, the industrial biotransformation was considered to proceed using fuel‐based resources to heat the factor (*F* = 0.7 kg_CO2_ kW‐h^−1^), while for photobiocatalytic reactions electricity is considered [158], using the European grid impact (*F* = 0.25 kg_CO2_ kW‐h^−1^), and the hydropower‐based one (*F* = 0.04 kg_CO2_ kW‐h^−1^). Estimated GWPs are depicted in Figure 7.

As observed, the main environmental impact (with respect to GWP) is derived from the wastewater treatment. Even at mild WWTP processing, the impact is considerable when diluted systems are established (~15 kg_CO2_ kg_product_^−1^), because large volumes of aqueous media are needed to afford one kilogram [77]. Conversely, the energy contribution to the GWP is relatively low, since processes are conducted at 30°C for 10 h (which seems realistic for industrial biotransformations). Overall, a classic industrial biotransformation under fuel‐based energy input at 100 g L^−1^ would lead to ~1 kg_CO2_ kg_product_^−1^ (upstream contribution), provided that a mild wastewater treatment can be conducted.

Therefore, to enable that the less impactful renewable energy or electricity could result environmentally on par with classic processes, photobiocatalytic systems should accumulate product titers in the range of ~75 g L^−1^. In absolute terms, those values appear unrealistic compared with current figures reported for photobiocatalysis (1–15 g L^−1^), due to the inherent product toxicity, which is detrimental to living cells. However, photobiocatalytic processes can still be (environmentally) advantageous in several scenarios, such as the use of discarded wastewater effluents as reaction media (i.e., containing polluting agents that would hamper the growth of other microorganisms or salty environments [66] or in processes where growing cells may provide benefits compared to resting cell biotransformations. Moreover, one should consider the CO_2_ uptake during the cyanobacteria growth (although such CO_2_ would be neutrally emitted again upon biomass mineralization).

Subsequently, the extractive downstream unit may have an environmental impact as well. At first sight, one could consider that the impact of the DSP would be identical in both cases (same product, same water media, same extraction). However, the required solvent volume depends on the product titer, with obviously larger volumes to be consumed in diluted systems [77]. Thus, with current values, photobiocatalytic systems would exert a larger environmental impact in the downstream, compared to an established industrial biotransformation. Solvent recovery and reuse or exploring other less impactful downstream units (e.g., crystallization) would improve the GWPs associated. Likewise, establishment of multi‐step processes that could save downstream steps, may be beneficial as well. All in all, process intensification and penetration of renewable energy (e.g., for wastewater treatment plants) appear to be the first aspects to be tackled.

## 7 Summary and Outlook

Photobiocatalysis has emerged as an important field of green chemistry, producing chemicals in high enantioselectivities. In particular, utilizing whole cells of photoautotrophic microorganisms such as cyanobacteria and microalgae to produce recombinant enzymes have allowed higher atom‐ and reaction mass efficiencies as compared to their heterotrophic counterparts. By coupling enzymes (particularly oxidoreductases) to natural photosynthesis, reactions can be driven via water oxidation, providing the reducing equivalents and, in some reactions, also photosynthetic oxygen.

Industrial application of photoautotrophic microorganisms still remains a challenge due to the limited cell densities allowed in batch reactors, mainly attributed to light attenuation. Several solutions with regards to reactor and material developments have been employed to alleviate this. Continuous micro‐ and meso‐reactors are now being developed and utilized to alleviate light shading, showing high STYs. Furthermore, matrices used for immobilizing or entrapping photoautotrophic microorganisms are also being developed from submicron alginate matrices to nanochitin with improved pore sizes aiding mass transfer in the matrix.

Other challenges in utilizing cyanobacterial or microalgal whole cells are their slow growth and the difficulty to express genes in their genome. This is now being circumvented by developing strains with a faster growth rate than the widely used *Synechocystis* sp. PCC 6803 and by employing systems which can shuttle electrons out of the cell toward the reaction of interest. Genetic toolboxes are also being expanded for fast growing strains to hasten gene introduction.

However, sustainability assessments reveal that product titers achieved using photobiocatalysis are still significantly lower as compared to classic processes. The main contributor is wastewater treatment, especially in diluted systems where significant aqueous volumes can generate large volumes of CO_2_ per kg of product. Utilizing wastewater streams such as saline environments can potentially be advantageous. This should also go hand‐in‐hand with efficient DSP, improved solvent recovery, alternative downstream strategies to reduce its overall environmental impact.

## Supporting Information

Additional supporting information can be found online in the Supporting Information section.

## Funding

This study was supported by Austrian Science Fund (10.55776/P 36614‐B, 10.55776/COE17).

## Conflicts of Interest

The authors declare no conflicts of interest.

## Supporting information

Supplementary Material

## Data Availability

Data sharing not applicable to this article as no datasets were generated or analysed during the current study.
