# Supplementary material for: Toward Sustainability: Intensification of Light‐Driven Whole Cell Biocatalysis
Source: Chembiochem. 2026 Apr 22;27(8):e70320. doi: 10.1002/cbic.70320 (PMC13101868; doi:10.1002/cbic.70320)
Supplement: Supplementary file 1 — Supplementary Material [file CBIC-27-e70320-s001.pdf]

Supporting Information

Towards Sustainability – Intensification of Light-Driven Whole Cell Biocatalysis

Lenny Malihan-Yap, [a] Pablo Domínguez de Maria, [b] and Robert Kourist*[a]

[a] Institute of Molecular Biotechnology, Graz University of Technology, Petersgasse 14, Graz 8010
[b] Sustainable Momentum, SL, Av. Ansite 3, 4-6, 35011 Las Palmas de Gran Canaria, Canary Islands, Spain

I. Wastewater Treatment Contribution to GWP (Upstream in Water)

Equation [1] is used to calculate for the contribution of wastewater treatment to global warming potential (GWP) using the data provided by Novartis for mild Wastewater treatment plant (WWTP) which is 0.073 kg CO_2 kg wastewater^{-1} and using the density of water (1.0 g cm^{-3}). For the review, it is assumed that the entire waste fraction is sent to WWTP, then the equation can be simplified to [2]

$$\mathrm{GWP}(\mathrm{water}(\mathrm{WWTP}))=\frac{0.073\cdot\%\mathrm{WaterTreated}}{100\cdot[\mathrm{P}]} \qquad [1]$$

$$G\mathrm{WP}(\mathrm{media})=\frac{0.073}{[\mathrm{P}]} \qquad [2]$$

Where, %WaterTreated – proportion of the wastewater that is sent to the treatment (e.g., if some proportion is recycled, no impact on GWP); [P] – product titer, kg L^{-1}

II. Energy Contribution to GWP (Upstream in Water)

The reactors are oftentimes heated and stabilized at a certain temperature. Thus, the energy required to heat a liquid depends on the specific heat capacity (Cp) of the liquid, and can be calculated according to Equation [3].

$$\mathrm{Q}=\mathrm{mC_p}\Delta\mathrm{T} \qquad [3]$$

The equation can be developed using the Cp of water (4.18 KJ kg^{-1} ºC^{-1}) and its density (1 g cm^{-3}) to equation [4]. Since the water volume used in the upstream to produce one kilogram of product can be estimated based on the product titers as 1/[P], where [P] is the product titer in kg L^{-1}.

$$\mathrm{Q}=\frac{4.18\cdot\Delta\mathrm{T}}{[\mathrm{P}]} \qquad [4]$$

Assuming 15% of extra energy for each hour of reaction to hold the required temperature, and considering the reaction time (t), in hours, the total energy needed for the upstream would be:

$$Q=\left(\frac{4.18\cdot\Delta T}{[P]}\right)+0.15\cdot t\cdot\left(\frac{4.18\cdot\Delta T}{[P]}\right) \qquad [5]$$

Subsequently, the energy (Q) must be converted in GWP. To that end, the overall obtained Q value is divided by 3600 to convert KJ into kWh^{-1}, and it is multiplied by the currently assumed Factor F (kg CO_2 · kWh^{-1}), which depends on the source of energy.

$$G\mathrm{WP}(\mathrm{water}(\mathrm{energy}))=\frac{1.25\cdot\mathrm{F}}{(3600)}\cdot\left[\left(\frac{4.18\cdot\Delta\mathrm{T}}{[\mathrm{P}]}\right)+0.15\cdot\mathrm{t}\cdot\left(\frac{4.18\cdot\Delta\mathrm{T}}{[\mathrm{P}]}\right)\right] \qquad [6]$$

$$\mathrm{GWP}(\mathrm{water}(\mathrm{energy}))=\mathrm{F}\cdot\left[\left(\frac{0.00145\cdot\Delta\mathrm{T}}{[\mathrm{P}]}\right)+\mathrm{t}\cdot\left(\frac{0.00022\cdot\Delta\mathrm{T}}{[\mathrm{P}]}\right)\right] \qquad [7]$$

Where, F is 0.7 kg CO_2 / kg product for fuel-based processes; F is 0.25 kg CO_2 / kg product for European grid electricity and F is 0.04 kg CO_2 / kg product for renewable electricity.
